# Whole Plastome Sequencing Within *Silene* Section *Psammophilae* Reveals Mainland Hybridization and Divergence With the Balearic Island Populations

**DOI:** 10.3389/fpls.2019.01466

**Published:** 2019-11-15

**Authors:** José Carlos del Valle, Inés Casimiro-Soriguer, Mᵃ Luisa Buide, Eduardo Narbona, Justen B. Whittall

**Affiliations:** ^1^Department of Molecular Biology and Biochemical Engineering, Pablo de Olavide University, Seville, Spain; ^2^Department of Biology, Santa Clara University, Santa Clara, CA, United States

**Keywords:** allopatric speciation, Balearic Islands, genome skimming, hybridization, Iberian Peninsula, introgression, Sileneae

## Abstract

Reconstructing the phylogenetic relationships within Caryophyllaceae tribe Sileneae has been obscured by hybridization and incomplete lineage sorting. *Silene* is the largest genus in the Caryophyllaceae, and unraveling its evolutionary history has been particularly challenging. In order to infer the phylogenetic relationships among the five species in *Silene* section *Psammophilae*, we have performed a genome skimming approach to acquire the complete plastid genome (cpDNA), nuclear ribosomal cistron (nrDNA), and partial mitochondrial genome (mtDNA). We have included 26 populations, representing the range of each species' distribution. This section includes five morphologically similar species endemic to the Iberian Peninsula and Balearic Islands (Ibiza and Formentera), yet some of them occupy distinct edaphic habitats (e.g. maritime sands, calcareous sandstones). In addition to phylogeographic analyses, genetic structuring using the chloroplast data set was inferred with Discriminant Analysis of Principal Components (DAPC), analyses of molecular variance (AMOVA), and a partial Mantel test. Reference-guided assembly of 50 bp single-end and 250 bp paired-end Illumina reads produced the nearly complete cpDNA genome (154 kbp), partial mtDNA genome (from 81 to 114 kbp), and the nrDNA cistron (6.4 kbp). Selected variable regions of the cpDNA and mtDNA assemblies were confirmed by Sanger sequencing. Phylogenetic analyses of the mainland populations reveal incongruence among the three genomes. None of the three data sets produced relationships consistent with taxonomy or geography. In contrast, *Silene cambessedesii,* present in the Balearic Islands, is the only species that forms a strongly supported monophyletic clade in the cpDNA genome and is strongly differentiated with respect to the remaining taxa of the Iberian Peninsula. These results contrast with those obtained for mainland populations. Across the entire analysis, only one well-supported mainland clade of *Silene littorea* and *Silene stockenii* emerges from the southern region of the Iberian Peninsula. DAPC and AMOVA results suggest the absence of genetic structure among mainland populations of *Silene* section *Psammophilae*, whereas partial Mantel test discarded spatial correlation of genetic differentiation. The widespread incongruence between morphology-based taxonomic boundaries and phylogeography suggests a history of interspecific hybridization, in which only a substantial geographic barrier, like isolation by the Mediterranean Sea, was sufficient to create and maintain species boundaries in *Silene* section *Psammophilae*.

## Introduction

The Mediterranean Basin is commonly described as one of the most important biodiversity hotspots in the world (Médail and Quézel, 1997; Médail and Quézel, 1999; Myers et al., 2000). In particular, the Iberian Peninsula and adjacent Balearic Islands emerge as key centers of biodiversity due to their complex geological history (including a great diversity of substrates such as serpentines, dolomites, and gypsum, among others) and spatially heterogeneous climate (Médail and Quézel, 1997; Thompson, 2005), making them ideal for examining biogeographic and evolutionary processes in plants. The coupling of the geographical position of the Iberian Peninsula (flanked by the Pyrenees to the north, the Atlantic Ocean to the west, and the Mediterranean Sea to the south and east) and Balearic Islands (isolated from mainland) with Mediterranean climate leads to exceptional ecological opportunity for habitat differentiation, geographic separation, and subsequent reproductive isolation (Thompson, 2005).

The Balearic Islands are especially rich in endemics, making them excellent models for understanding speciation (e.g. Juan et al., 2004). The Balearic archipelago was separated from the mainland in the Oligocene [30–25 million years ago (Ma)], yet ephemeral land bridges connecting the mainland to the islands formed during the Messinian Salinity Crisis in the Late Miocene (ca. 5.5 Ma) (Hsü et al., 1973; Krijgsman et al., 1999; Duggen et al., 2003). Due to long-term isolation after the flooding of the Mediterranean Sea, biota on these islands gradually developed into locally adapted, novel species. The scarcity of new taxa coming to the Balearic Islands after their isolation contrasts with the colonization events in other Mediterranean islands. In the Aegean islands, for instance, glaciations during the Late Pleistocene (∼0.8–0.01 Ma) decreased sea levels and created land bridges that allowed the colonization of many mainland taxa (e.g. *Nigella arvensis* and *Silene gigantea* complexes, dwarf elephants, or pigmy hippopotami, among others) (Reyment, 1983; Bittkau and Comes, 2009; Du Pasquier et al., 2017), whereas Balearic Islands remained isolated because no land bridges connected them to the mainland during this time (Van der Geer et al., 2010).

In the Iberian Peninsula, dramatic geological and climatic changes (e.g. Quaternary glaciations) have repeatedly caused fragmentation, contraction, and expansion of species ranges. In this context, recently diverged lineages may have experienced secondary contact and increased chances for hybridization and introgression (Thompson, 2005; Nieto Feliner, 2014). Hybridization is a prominent force in plant evolution that allows them to acquire genetic novelties faster than through mutations alone, creating opportunities for adaptive evolution (Arnold, 1997; Rieseberg, 1997; Rieseberg et al., 2003; Mallet, 2005). Introgressive hybridization, whether it be adaptive or neutral, may distort phylogenetic relationships in plant species where reproductive isolation is incomplete. In plants, molecular studies have tried to overcome this issue by using chloroplast sequences in addition to information from nuclear DNA sequences. Hence, incongruences between nuclear- and chloroplast-based phylogenetic trees are frequently interpreted to be the result of introgressive hybridization (e.g. Soltis and Kuzoff, 1995; Okuyama et al., 2005; Wang et al., 2016). However, phylogenetic incongruences can also be caused by incomplete lineage sorting, especially when the speciation is rapid, recent, and without persistent bottlenecks (Frajman et al., 2009a). Disentangling hybridization and incomplete lineage sorting long inhibited interpretation of incongruent molecular data sets, yet this is a matter of ongoing research, and several methods have been developed in recent years to differentiate these two historical processes (e.g. Holland et al., 2008; Joly et al., 2009).

In the tribe Sileneae (Caryophyllaceae), ancient and recent hybridization events have been proposed to be important processes that must be considered when inferring phylogenetic relationships (Erixon and Oxelman, 2008). Several studies have stressed the importance of hybridization to understand the evolutionary history of many groups within Sileneae (e.g. Erixon and Oxelman, 2008; Rautenberg et al., 2010; Petri and Oxelman, 2011; Petri et al., 2013). The ability of *Silene latifolia* and *Silene dioica* to hybridize is one of the best examples of incomplete reproductive isolation in this group (Bernasconi et al., 2009). These two closely related species show strong differences in their morphology and ecological preferences (e.g. *S. latifolia* has white flowers and grows in dry and disturbed habitats, whereas *S. dioica* has red flowers and inhabits moister soils), but these two lineages hybridize when in sympatry (Baker, 1948). Thus, their ecological and morphological differences suggest they are unique lineages, yet the lack of genetic differentiation in sympatry is the one hallmark of introgressive hybridization (Minder et al., 2007; Hathaway et al., 2009).

Inferring phylogenetic relationships within Sileneae is complicated, and the resulting phylogenies are frequently incongruent with morphology-based classifications (Oxelman and Lidén, 1995; Oxelman et al., 1997; Oxelman et al., 2001; Frajman et al., 2009b; Naciri et al., 2017). The tribe Sileneae is subdivided into eight genera (*Agrostemma* L., *Atocion* Adans., *Eudianthe* (Rchb.) Rchb., *Heliosperma* Rchb., *Lychnis* L, *Petrocoptis* A. Braun ex Endl., *Silene* L., and *Viscaria* Bernh.) (Oxelman et al., 2001), of which the genus *Silene* is the most diverse, with approximately 470 species (Oxelman et al., 2013; Petri et al., 2013), although some studies suggest up to 700 species (e.g. Greuter, 1995). Several phylogenetic studies subdivided this genus into two well-supported subgenera: subgenus *Silene* and subgenus *Behenantha* (Popp and Oxelman, 2004; Popp and Oxelman, 2007; Erixon and Oxelman, 2008; Frajman et al., 2009a; Rautenberg et al., 2010; Rautenberg et al., 2012). However, these analyses based on chloroplast loci, nuclear ribosomal regions, and low-copy nuclear DNA led to unresolved phylogenetic relationships within each subgenus, probably due to ancient and recent hybridization (e.g. Frajman and Oxelman, 2007; Erixon and Oxelman, 2008; Rautenberg et al., 2010). Hybridization has been documented in several groups within *Silene*, for instance, in *Silene* section *Physolychnis* (Petri and Oxelman, 2011), Section *Melandrium* (Rautenberg et al., 2010), and Section *Otites* (Balounova et al., 2019), as well as in polyploid *Silene* from North America (Popp and Oxelman, 2007). Yet, the more remarkable event is the introgression between species from distinct subgenera about 6.6 Ma after the divergence of the two subgenera had occurred (Petri et al., 2013).

**Figure 1:**
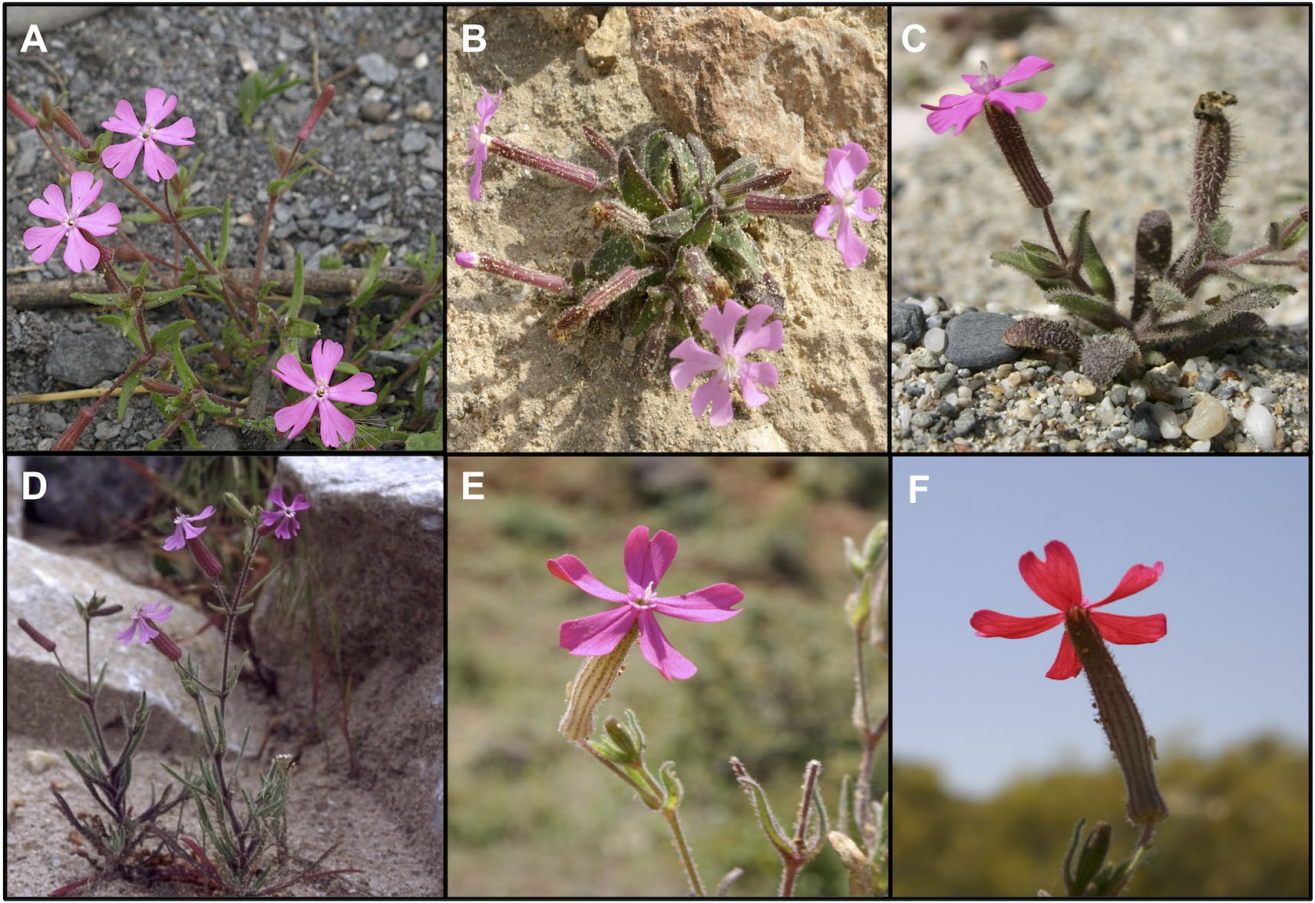
Photographs of the five species belonging to *Silene* section *Psammophilae*. **(A)** *Silene adscendens*, **(B)** *Silene cambessedesii*, **(C)** *Silene littorea*, **(D)** *Silene psammitis*, and **(E**, **F)** *Silene stockenii* (showing the characteristic upper and lower side of petals).

*Silene* section *Psammophilae* (Talavera) Greuter is a monophyletic group within the subgenus *Behenantha* that is composed of five species endemic to the Iberian Peninsula and Balearic Islands: *Silene adscendens* Lag., *Silene cambessedesii* Boiss. & Reut., *Silene littorea* Brot., *Silene stockenii* Chater, and *S. psammitis* Link ex Spreng. (Oxelman et al., 2013) (**Figure 1**). This section was previously considered a subsection within section *Erectorefractae* Chowdhuri (Talavera, 1979). However, Greuter (1995) proposed the sectional status for subsection *Psammophilae* based on differences in cell shape and flowering time with respect to other members of the section *Erectorefractae*, in addition to the differences previously described by Talavera et al. (1979) (e.g. monochasium inflorescences in *Psammophilae* and dichasium in *Erectorefractae*). *Silene pendula* L. was also previously included in *Silene* section *Psammophilae*, but it was placed in section *Behenantha* Otth by Oxelman et al. (2013). In addition, analysis based on ITS supports the monophyly of *S. adscendens*, *S. cambessedesii*, *S. littorea*, *S. psammitis*, and *S. stockenii* (Casimiro-Soriguer, 2015).

**Figure 2:**
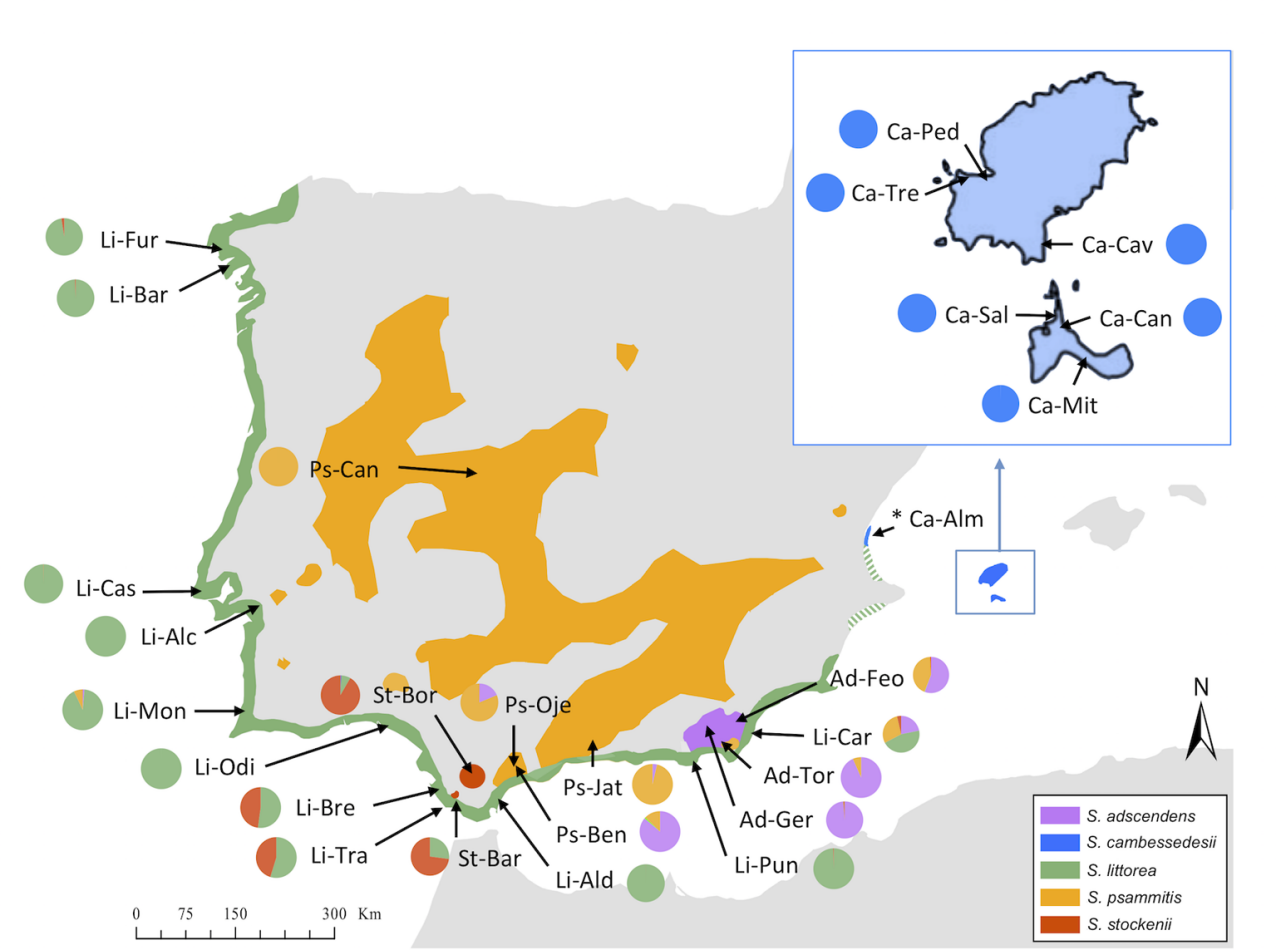
Geographical distribution of studied populations of *Silene* section *Psammophilae* in the Iberian Peninsula and Balearic Islands and Discriminant Analysis of Principal Components (DAPC) results. Colored areas represent the distribution area of each species. The colored pie graphs represent the membership probability of each species according to the DAPC analysis on the complete plastid genome (cpDNA) when using species categories as priors. Species and population codes are shown in **Table 1**.

The basic chromosome number of species of the Section *Psammophilae* is n = 12, and the two species with available information are diploids, 2n = 24 (Talavera, 1990). They are self-compatible and are mainly pollinated by insects, although low levels of autonomous self-pollination may exist. They are all annual species, glandular-pubescent, with the inflorescence consisting of a monochasial cyme; but they differ in seed-coat ornamentation and in the length of the calyx and carpophore in fruit (Talavera, 1990; Casimiro-Soriguer, 2015). In addition to differences in their phenotypic traits, these five species have non-overlapping geographical distributions and distinct edaphic affinities. *S. littorea* grows in coastal sandy substrates along a coastal fringe from the northwestern to southeastern regions of the Iberian Peninsula. *S. cambessedesii* occurs in the same habitat on the Mediterranean islands of Ibiza and Formentera (the two largest western islands of the Balearic Islands), but it is also known from a few populations on the east coast of the Iberian Peninsula (Almenara, Comunidad Valenciana). *S. adscendens* occurs on intermittent streams of the southeastern Iberian Peninsula. *S. stockenii* grows in calcareous sandstones and is an endangered species restricted to the southern end of the Iberian Peninsula. Finally, *S. psammitis* is distributed throughout the Iberian Peninsula on granite or slates (subsp. *psammitis*) or dolomitic sands, and rarely on clay and serpentine (subsp. *lasiostyla*) between 300 and 1,500 m (**Figure 2**) (Talavera, 1979).

Here, we aim to unravel the phylogenetic relationships of species in *Silene* section *Psammophilae* using next-generation DNA sequencing. Recent improvements in DNA sequencing have made it possible to sequence nearly complete organellar genomes from genomic DNA (genome skimming), which has helped to infer organismal phylogenies at low taxonomic levels across different groups of angiosperms (e.g. Parks et al., 2009; Whittall et al., 2010; Kane et al., 2012; Ruhsam et al., 2015). In this study, we will address the following questions: Can genome skimming resolve the relationships of mainland and island species of *Silene* section *Psammophilae*? Do the relationships align with morphology-based species boundaries, or do they reflect geographic distance? Is genetic differentiation among these lineages correlated with geography (specifically between mainland and island populations)? Can biogeographic events (i.e. Messinian Salinity Crisis) explain the colonization of Balearic Islands by the island species of this section based on the estimating timing of mainland–island divergence? We employ sequence data from the complete plastome (cpDNA), nuclear ribosomal cistron (nrDNA), and partial mitochondrial genome (mtDNA) to address these questions using phylogenetic analysis and population genetics.

## Materials and Methods

### Sampling, Genomic DNA Extraction, and Sequencing

Fresh leaf samples were collected in 2010–2012 from a total of 26 natural populations spanning the geographical range of species in *Silene* section *Psammophilae* (**Figure 2**). For each population, the DNA of five individuals was extracted using DNeasy Plant Mini Kit (Qiagen, Valencia, USA) and pooled into a single sample. DNA concentration and purity were measured with a NanoDrop ND-1000 spectrophotometer (NanoDrop Technologies, Inc., Wilmington, USA). Total genomic DNA was used to prepare next-generation sequencing libraries following the Nextera kit (Illumina, San Diego, USA), barcoded with 6 (single-end reads) and 8 (paired-end reads) bp indices, and sequenced at the Epigenome Center of the University of Southern California. Four libraries were pooled in equimolar concentrations and shared a half lane of an Illumina HiSeq 2000 in which they were sequenced with 50 bp single-end reads. Fortuitously, these four libraries were sequenced twice. The remaining 22 libraries were indexed and then sequenced on a single lane on an Illumina HiSeq 2500 (San Diego, USA), which produced 250 bp paired-end reads.

### Reference-Guided Assemblies (cpDNA, mtDNA, and nrDNA)

Prior to assembly, 3' and 5' ends of sequences with more than a 5% chance of an error per base were trimmed in Geneious v.8.1.6 (Biomatters Ltd., Auckland, New Zealand) to remove low-quality regions. Then, we conducted a reference-guided assembly using the cpDNA and mtDNA genomes of *S. latifolia* as the reference (GenBank accession numbers NC_016730 and NC_014487, respectively) and a chimeric *Silene* nrDNA cistron (see details below). We used the Geneious assembler under default settings with medium-low sensitivity and 10 iterations (Kearse et al., 2012). Regions with less than 5× sequence coverage were considered as missing data. A consensus sequence was generated for each population using an 85% consensus threshold. Thus, ambiguity codes were applied for sites below 85% consensus arising from sequencing errors or due to a variable site in the pool of five individuals per population, assuming approximately equal sequencing coverage among the five individuals pooled per sample. Annotations were transferred using a 75% similarity cutoff to the reference genome. Sequences were aligned using the MAFFT (Katoh and Standley, 2013) plugin within Geneious with default settings. Finally, regions of questionable alignment were manually adjusted or masked before subsequent analyses.

No complete nrDNA cistron sequence was available as a reference from any single species of *Silene*. Thus, we created a chimeric reference sequence following a similar procedure described in Ripma et al. (2014). We downloaded from GenBank the complete 18S (1,733 bp; AF207027) and 28S (3,332 bp; AF479084) from *Stellaria media*, the closest relative with complete sequences for these regions. We combined these with the 5.8S and both internal transcribed spacer regions (ITS1 and ITS2) from *S. littorea* (832 bp; FN821094). In addition, we performed Sanger sequencing to obtain the complete 5.8S gene with both internal transcribed spacer regions from a subsample of *Silene* section *Psammophilae* (Casimiro-Soriguer, 2015). All aforementioned nrDNA sequences were aligned, and the consensus sequence was extracted following the same settings previously described for the cpDNA. The resulting sequence was used as the nrDNA reference during the reference-guided assembly process following the parameters described above.

### Single Nucleotide Polymorphism Validation

Sanger sequencing was performed in order to validate putative SNPs (single nucleotide polymorphism) that were discovered after aligning the next-generation sequencing data. Since each population was represented by a pool of five individuals, ambiguities could represent genetic variation within the pool. Thus, we individually amplified and sequenced as many individuals as possible from several of the population pools. Within each genome, we selected a specific region with the highest number of phylogenetically informative sites. For the cpDNA, we designed primers specific to the *trnK* region to amplify and sequence an 814 bp fragment that spanned 60 putative SNPs across 26 samples (*trnK-F*: GCTCGTTGCTTATTCTTTCCACA and *trnK-R*: ACTTTTGTTGGATTGGCGCT). For the mtDNA, primers were designed for the *atp1* region in order to amplify a 753 bp fragment with 125 putative SNPs (*atp1-F*: GAGTCGCAGCATCAAGGTCT and *atp1-R*: GCGGTAGATAGCCTGGTTCC). PCR conditions followed those of Dick et al. (2011) using *Taq* polymerase (New England Biolabs, Ipswich, USA) with the following thermal cycling steps: initial denature at 95°C for 3 min; 35 cycles of 95°C for 30 s, 50°C (*trnK*) and 67°C (*atp1*) for 30 s, 72°C for 90 s, a final extension at 72°C for 10 min, and a 4°C hold. PCR products were purified using exoSAP (Thermo Fisher, Cleveland, USA) and sequenced using Big Dye Terminator methodology on an ABI 3730xl DNA Analyzer (Sequetech Corp., Mountain View, USA). Single contigs were created by aligning forward and reverse reads. Contigs were then aligned to the next-generation sequences in Geneious to determine the validity of the putative SNPs.

### Phylogenetic Analyses

Phylogenetic reconstruction was performed using both maximum likelihood (ML) and Bayesian approaches in RAxML (Stamatakis, 2014) and MrBayes, respectively (Huelsenbeck and Ronquist, 2001). For the ML analysis, we used the GTR+CAT approximation of the GTR+G model of nucleotide evolution with estimate of proportion of invariable sites and 1,000 bootstrap replicates. For the Bayesian analysis, we applied the GTR+G+I model of nucleotide evolution for two separate runs, each consisting of four independent chains run for 10,000,000 generations sampling every 50,000 generations after 1,000,000 generations of burn-in. Bayesian analysis runs were checked for proper mixing and convergence using Tracer v.1.6 (Rambaut et al., 2014). The cpDNA-based tree was rooted using the complete genomes of *S. latifolia* and *Silene vulgaris* (GenBank accession numbers NC_016730 and NC_016727). The low sequencing depths and subsequent assembly challenges for the mtDNA genome limited the number of sites that could be unambiguously aligned (see *Results*). Therefore, we selected six mitochondrial coding regions representing a range of substitution rates previously used in *Silene* (Barr et al., 2007; Sloan et al., 2008; Sloan et al., 2009; Rautenberg et al., 2012): the protein-encoding ATP synthase subunit 1 (*atp1*), ATP synthase subunit 4 (*atp4*), ATP synthase subunit 6 (*atp6*), cytochrome b (*cob*), cytochrome c oxidase subunit 3 (*cox3*), and NADH dehydrogenase subunit 9 (*nad9*). The mtDNA-based tree was rooted using a concatenation of these six mitochondrial genes from *S. latifolia* (extracted from NC_014487) and *S. vulgaris* (extracted from chromosomes 1 and 2 of mtDNA genome; JF750427 and JF750429, respectively). The nrDNA-based tree was rooted using the ITS regions of *S. latifolia* and *S. vulgaris* (FJ384022 and KJ918500, respectively). The resulting trees were visualized using FigTree v.1.4.2 (Rambaut, 2014). Bootstrap values (BSs) ≥ 85/posterior probabilities (PPs) ≥ 0.90 were considered as strong support, while values of 70–85% BS and 0.80–0.90 PP were considered as moderate support. In addition to the ML and Bayesian analyses, we explored phylogenetic uncertainly in the cpDNA with a NeighborNet network in SplitsTree 4 v. 4.13.1 (Huson and Bryant, 2006) with uncorrected P distances and ambiguous sites treated with the "Average States" option.

### Gene Tree–Species Tree Reconciliation

A species tree was estimated in *BEAST (Heled and Drummond, 2010) implemented in BEAST v.2.4.2 (Bouckaert et al., 2014). One limitation of *BEAST is the coalescence requirements based on which you should assign each sample to one of the five morphologically defined species. Although this could produce meaningless results if the morphologically defined species do not exist, we are confident that the morphological and ecological uniqueness of these lineages justify such *a priori* assignments. We used two partitions of the cpDNA genome with linked genealogies: the third codon position and non-coding regions of cpDNA, and the first and second codon position of the coding regions of the cpDNA. MCMC analysis was run for 500 million generations, sampling every 50,000 generations, using Yule speciation tree prior and the most appropriate nucleotide substitution model for each partition as chosen by jModelTest v. 2.1.10 (Darriba et al., 2012) under the Akaike information criterion (AIC). The selected nucleotide substitution models were GTR+G+I for both the third codon position and non-coding regions of the cpDNA and for the first and second codon position of the coding regions of the cpDNA. We used an uncorrelated log-normal relaxed molecular clock and the estimated separation of *S. latifolia* and *S. vulgaris* as 5.36 Ma (Frajman et al., 2009a) with a normally distributed standard deviation of 1.0, as calibration. The first 10% of trees were used as burn-in. Convergence and mixing were assessed in Tracer v.1.6 (Rambaut et al., 2014), with all ESS values above 120. Trees were summarized in a maximum clade credibility tree using TreeAnotator v.2.4.2 (Drummond et al., 2012). Species trees were visualized in DensiTree v.2.2.5 (Bouckaert and Heled, 2014).

### Population Genetic Structure

Population structuring within the *Silene* section *Psammophilae* was explored using variable sites of the whole cpDNA genome. Since the cpDNA data set had the most variable sites (>24×) and the strongest signal (cpDNA phylogeny has more than four times the number of nodes with strong support compared to mtDNA or nrDNA), we have only analyzed the cpDNA at the population level. We used the Discriminant Analysis of Principal Components (DAPC) (Jombart, 2008; Jombart et al., 2010) to study population subdivision. We ran two clustering analyses to assess introgression among populations using both the species categories and locations as prior categories. We used species categories as priors to test the likelihood of correct assignment of populations to each species, whereas using locations as priors, we tested whether populations cluster by their geographical proximity. The number of principal components was set according to an alpha-score optimization (i.e. trade-off between power of discrimination and overfitting) (Jombart and Collins, 2015). DAPC analyses were performed using the "adegenet" package v.2.0.0 (Jombart, 2008) for the R software v.3.2.3 (R Core Team, 2016). Population structuring was explored using the "clustering with linked loci" option implemented in BAPS v.6.0 (Corander et al., 2008), which unlike STRUCTURE allows for linked loci. The number of genetically homogeneous groups was estimated from the PP (log marginal likelihood of the best partition) for three iterations of *K* = 1–26 (the total number of populations).

We tested for a correlation between genetic variation in the cpDNA genome and geographic distance between populations using a partial Mantel test (Mantel, 1967). The analysis was performed in R software v.3.2.3 (R Core Team, 2016) using the "vegan" package v.2.5.2 (Oksanen et al., 2018), with 100,000 permutations to test for significance. Tests were performed on all populations, as well as separately on just the mainland and just the island populations to dissect the relative contributions of these two groups on any isolation-by-distance findings. Additionally, analyses of molecular variance (AMOVA) (Excoffier et al., 1992) were conducted using Arlequin v.3.5 (Excoffier and Lischer, 2010). An AMOVA was conducted to assess genetic differentiation in the cpDNA genome among all studied species. A second AMOVA with just mainland species was performed to exclude the influence of island populations in the analysis. Finally, a third AMOVA was carried out to study genetic differentiation among islands. *F*-statistics (Wright, 1951) were used to estimate the proportion of genetic differentiation found among species, with significant levels determined by 1,023 permutations.

### Similarity of *S. cambessedesii* Plants From Mainland and Balearic Populations

We assessed genetic similarities of *S. cambessedesii* from Balearic Islands with plants from a mainland population from the Iberian Peninsula (Almenara; **Figure 2**). Almenara is the only remaining natural population of *S. cambessedesii* on the mainland; however, plants from this population were not collected during the initial sampling for next-generation sequencing given their endangered status in the region (Comunidad Valenciana government) (Navarro et al., 2015). DNA was obtained from 20 seeds (provided by the Servicio de Vida Silvestre-CIEF in Valencia, Spain) of botanical garden grown plants from this population. Since we received seeds after the preparation of the next-generation sequencing libraries, we amplified and sequenced only four regions representing the three genomes. From the chloroplast genome, we amplified the *trnK* (see previous section *Single Nucleotide Polymorphism Validation*) and *ycf1* (871 bp) regions (using the primers *ycf1-F*: CAGTTTTTCCATTGAGTCCGTCC and *ycf1-R*: TCCCGAAAACGACCCCATTT). From the mitochondrial genome, we amplified and sequenced a fragment of the *atp1* gene (see previous section *Single Nucleotide Polymorphism Validation*). From the nuclear genome, we amplified and sequenced the ITS region (using the primers ITS5* and *ITS26S-25R* as in Whittall et al., 2006). PCR conditions and purification were the same previously described, but using 55°C and 59°C annealing temperature for ITS and *ycf1* regions, respectively.

Finally, we examined the phylogenetic relationships between mainland and island individuals of *S. cambessedesii* by building ML trees in RAxML for each fragment of the cpDNA (*trnK* and *ycf1*), mtDNA (*atp1*), and nrDNA (ITS) genomes. ML analyses were performed using the GTR+CAT approximation of the GTR+G model of nucleotide evolution with estimate of proportion of invariable sites and 1,000 bootstrap replicates. ML trees were rooted using *S. latifolia* and *S. vulgaris* as outgroups. The *trnK*, *ycf1*, and *atp1* fragments were extracted from the complete chloroplast and mitochondrial genomes of *S. latifolia* (NC_016730 and NC_014487 for cpDNA and mtDNA genomes, respectively) and *S. vulgaris* (NC_016727 and JF750427 for cpDNA and mtDNA genomes, respectively), whereas the ITS regions of *S. latifolia* and *S. vulgaris* were obtained from GenBank (FJ384022 and KJ918500, respectively).

## Results

### Genomic DNA Extraction and Sequencing

Single-end sequencing generated two data sets that were merged since similar results were attained from each individual data set, obtaining an average of 25.91 million reads per sample (range 24.3 million—28.0 million; **Supplementary Table S1**). Of those, approximately 1.70 million raw reads (6.6%) were trimmed prior the assemblies. Paired-end sequencing provided an average of 1.47 million reads per sample (range 0.79 million–2.22 million; **Supplementary Table S1**). An average of approximately 60,000 raw reads were trimmed from each sample (4.0%). Raw data are available from GenBank's Short Read Archive (accession number PRJNA558348). GenBank accession numbers for cpDNA, mtDNA, and nrDNA genomes are available in **Supplementary Table S2**.

### Reference-Guided Assemblies (cpDNA, mtDNA, and nrDNA)

For the cpDNA, a nearly complete chloroplast assembly from each population was recovered (alignment length = 154,199 bp). Due to alignment ambiguities, we masked ∼1% of the total length (average = 1,535.6 bp; range 1,444–1,606 bp). Total cpDNA sequencing depths were between 49.9X and 2,064.8X, with a mean of 359.91X (median = 153.95X; **Supplementary Table S1**), and there were 6,322 variable sites not including the outgroup samples. For the mtDNA genome, a mitochondrial sequence from each sample was recovered (alignment length = 254,270 bp). However, on average, only 38.0% of the alignment was assembled for each individual sample (range 31.8–44.9%), mainly corresponding to coding regions. Sequencing depths were lower than for the cpDNA, ranging from 3.8X to 197.2X (mean = 33.4X; median = 10.5X; **Supplementary Table S1**). For each sample, a concatenation of 5,648 bp from six mitochondrial genes with high sequencing depths (*atp1*, *atp4*, *atp6*, *cob*, *cox3*, and *nad9*) was selected for phylogenetic analyses. For the mtDNA data, there were 130 variable sites not including the outgroup samples. For the nrDNA, we assembled the complete cistron sequence for all samples, including a portion of the external transcribed spacer (*ETS*) and non-transcribed spacer (*NTS*) regions. The length of the alignment was 6,415 bp, with sequencing depths between 885.8X and 7,584.7X and a mean depth of 2,765.2X (median = 2,046.7X; **Supplementary Table S1**). There were 257 variable sites not including the outgroup samples in the nrDNA alignment.

### SNP Validation

In order to confirm some of the next-generation sequencing SNPs detected in the alignments, we amplified and Sanger sequenced 30 individuals for the *trnK* gene of the cpDNA genome (alignment length = 806 bp). We Sanger sequenced 16,648 bp to compare with the next-generation sequences. A total of 16,502 bp (99.1%) agreed with those obtained during the next-generation sequencing and assembly process. Ninety-four base pairs (0.56%) correspond to ambiguities in the next-generation sequencing that were only partially confirmed because we could not amplify all individuals pooled for that population sample (i.e. we detected one of the bases that cause the ambiguity, but not the other). The remaining 52 bp (0.31%) could not be confirmed due to recalcitrant amplification of some DNA samples. Of the 60 putative SNPs present in the *trnK* gene, 3 SNPs (5.0%) were completely validated, 47 SNPs (78.3%) had ambiguities that were partially confirmed, and 10 SNPs (16.7%) could not be verified because of failed PCR reactions. Sequences obtained from Sanger sequencing did not reveal any incongruences with those obtained during the next-generation sequencing.

We also amplified and Sanger sequenced 28 individuals for the *atp1* gene of the mtDNA genome (alignment length = 753). In total, 15,813 bp of this region were compared with the next-generation sequences. We confirmed the veracity of 15,509 bp (98.1%), whereas 213 bp (1.35%) were partially validated (i.e. one of the two possible bases were identified at an ambiguous position), and 91 bp (0.58%) could not be confirmed due to failed PCR reactions. Of the 125 putative SNPs present in this gene, 11 SNPs (8.8%) were confirmed, 98 SNPs (78.4%) contained at least one ambiguity that was partially validated, and 16 SNPs (12.8%) could not be verified because of failed PCR reactions. Sequences obtained from Sanger sequencing did not reveal incongruences with those obtained during the next-generation sequencing.

The variability within populations (i.e. ambiguities resulting of pooling five individuals in each population) in both the *trnK* and the *atp1* regions was assessed by amplifying and Sanger sequencing two or more individuals per population. When two individuals per population were sequenced, genomic polymorphism was verified in 2 of 21 cases (9.5%), and ambiguities found in next-generation sequencing were partially validated in the remaining cases (90.5%). When three or four individuals per population were sequenced, we confirmed the population genomic polymorphism in 24 of 29 cases (82.8%) and performed partial validations of the ambiguities found in next-generation sequencing in the remaining cases (17.2%). Failed PCR reactions precluded our ability to sequence all five individuals of any population.

### Phylogenetic Analyses

**Figure 3:**
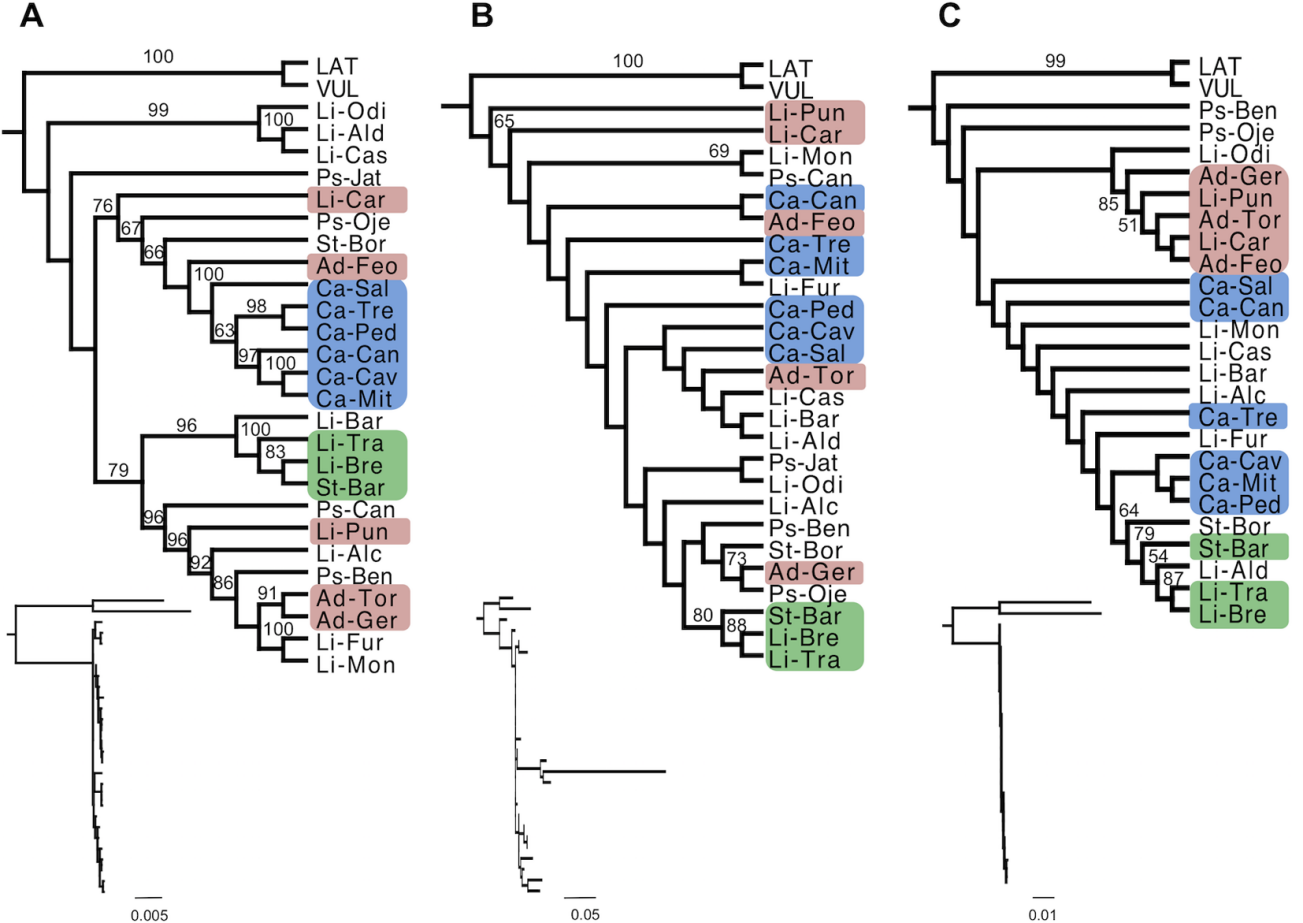
Phylogenetic relationships within the Section *Psammophilae* using maximum likelihood (ML) estimation. Phylogenetic relationships were determined from the complete chloroplast genomes **(A)**, six coding regions of the mitochondrial genome **(B)**, and complete nuclear ribosomal cistron with a partial ETS **(C)**. Sequences were aligned, rooted with two outgroups, and analyzes under maximum likelihood (ML) phylogenetic methods (RAxML). Numbers above branches represent bootstrap support (BS > 50 are displayed). Cladograms show relationships among taxa, while branch lengths are displayed in the inset phylograms. Branches are drawn proportional to the number of substitutions per site (see scale bar). Blue, green, and red squares represent populations from the Balearic Islands and the southern (Cadiz province) and southeast (Almeria province) of the Iberian Peninsula, respectively. Species and population codes are shown in **Table 1**.

ML and Bayesian phylogenetic analysis of the cpDNA genome showed mostly congruent topologies (**Figure 3A** and **Supplementary Figure S1A**). In the ML analysis, 17 of 27 (63%) internal branches were moderately or highly supported, while in the Bayesian analysis, 20 of 27 (74%) branches showed strong support. There are very few geographic patterns, and most species are not reciprocally monophyletic. An exception to this overall pattern is the six populations of *S. cambessedesii* from the Balearic Islands which form a strongly supported clade (BS = 100; **Figure 3A**). On the mainland, three geographically adjacent populations of *S. littorea* and *S. stockenii* (Li-Bre, Li-Tra, and St-Bar; see species and population codes in **Table 1**) from Cadiz province, in the southern end of the Iberian Peninsula, form another strongly supported clade (BS = 100). The network analysis of the cpDNA data produced a largely unresolved "starburst" with some differentiation of the Balearic Island samples combined with some mainland samples (**Supplementary Figure S2A**).

**Table 1:** Sample localities (populations of each species are ordered by name) and distance to the closest species.

Code	Species	Locality information	Latitude (°)	Longitude (°)	Distance to closest spp. (km)	Closest spp.
Ad-Feo	*Silene adscendens*	Spain, Almería, Los Feos	37.013444	−2.029278	13	*S. littorea*
Ad-Ger	*S. adscendens*	Spain, Almería, Gerjal	37.083361	−2.507861	20	*S. psammitis*
Ad-Tor	*S. adscendens*	Spain, Almería, Los Toros	36.822639	−2.043222	19	*S. littorea*
Ca-Can	*Silene cambessedesii*	Spain, Formentera, Canyes	38.729528	1.451861	184	*S. littorea*
Ca-Mit	*S. cambessedesii*	Spain, Formentera, Mitjorn	38.684389	1.467500	184	*S. littorea*
Ca-Sal	*S. cambessedesii*	Spain, Formentera, Ses Salines	38.746806	1.432889	183	*S. littorea*
Ca-Cav	*S. cambessedesii*	Spain, Ibiza, Cavallet	38.848139	1.401056	184	*S. littorea*
Ca-Ped	*S. cambessedesii*	Spain, Ibiza, Sa Pedrera	38.970028	1.261111	180	*S. littorea*
Ca-Tre	*S. cambessedesii*	Spain, Ibiza, Punta des Trencs	38.969194	1.270722	179	*S. littorea*
Li-Mon	*Silene littorea*	Portugal, Faro, Monte Clérigo	37.341174	−8.852668	95	*S. psammitis*
Li-Cas	*S. littorea*	Portugal, Lisboa, Cascais	38.702153	−9.473942	85	*S. psammitis*
Li-Alc	*S. littorea*	Portugal, Setúbal, Alcácer do Sal	38.485790	−8.903009	25	*S. psammitis*
Li-Fur	*S. littorea*	Spain, A Coruña, Furnas	42.638420	−9.039037	210	*S. psammitis*
Li-Car	*S. littorea*	Spain, Almería, Carboneras	36.962500	−1.899722	13	*S. adscendens*
Li-Pun	*S. littorea*	Spain, Almería, Punta Entinas	36.710261	−2.639618	7	*S. adscendens*
Li-Bre	*S. littorea*	Spain, Cádiz, Breña	36.189620	−5.949146	7	*S. stockenii*
Li-Tra	*S. littorea*	Spain, Cádiz, Trafalgar	36.182506	−6.034710	13	*S. stockenii*
Li-Odi	*S. littorea*	Spain, Huelva, Odiel	37.164706	−6.919111	45	*S. psammitis*
Li-Ald	*S. littorea*	Spain, Málaga, Aldea Beach	36.332278	−5.239083	16	*S. psammitis*
Li-Bar	*S. littorea*	Spain, Pontevedra, Barra	42.259707	−8.840256	180	*S. psammitis*
Ps-Can	*Silene psammitis*	Spain, Ávila, Candeleda	40.215608	−5.247733	300	*S. littorea*
Ps-Jat	*S. psammitis*	Spain, Granada, Játar	36.916194	−3.905028	40	*S. littorea*
Ps-Ben	*S. psammitis*	Spain, Málaga, Benahavis	36.511000	−5.035750	27	*S. littorea*
Ps-Oje	*S. psammitis*	Spain, Málaga, Ojen	36.592972	−4.857389	18	*S. littorea*
St-Bar	*Silene stockenii*	Spain, Cádiz, Barca de Vejer	36.247929	−5.914718	7	*S. littorea*
St-Bor	*S. stockenii*	Spain, Cádiz, Bornos	36.818347	−5.767805	65	*S. psammitis*

Topologies for mtDNA-based trees were mostly congruent for ML and Bayesian phylogenetic analysis (**Figure 3B** and **Supplementary Figure S1B**), yet only a few internal branches were confidentially resolved. In the ML analysis, 4 of 27 (15%) internal branches were moderately or highly supported, while in the Bayesian analysis, 6 of 27 (22%) branches showed moderate or strong support. The mtDNA-based trees did not show any clear phylogeographic pattern. Neither island nor mainland populations clustered geographically, except for the three adjacent populations of Cadiz province (Li-Bre, Li-Tra, and St-Bar) that formed a well-supported clade (BS = 88). The network analysis of the mtDNA data produced largely unresolved splits that do not correspond to clear taxon or geographical delimitations. In addition, the *S. littorea* from Barra (Li-Bar) had an exceptionally long terminal branch (**Supplementary Figure S2B**).

For the nrDNA genome, ML and Bayesian phylogenetic analysis were mostly congruent (**Figure 3C** and **Supplementary Figure S1C**), although very few internal branches were resolved. In the ML analysis, 4 of 27 (15%) internal branches were moderately or highly supported, while in the Bayesian analysis, only 4 of 27 (15%) branches showed moderate or strong support. Phylogenetic relationships in the nrDNA-based trees did not reflect either taxonomic boundaries or biogeographic patterns. The same three adjacent populations of Cadiz province (Li-Bre, Li-Tra, and St-Bar), together with a southern population of *S. littorea* (Li-Ald) separated approximately 65 km from these populations, formed a moderate supported clade (BS = 79). A strongly supported clade (BS = 85) emerged in the southeast of the Iberian Peninsula (Almeria province), composed by five adjacent populations: three populations of *S. adscendens* (Ad-Feo, Ad-Ger, and Ad-Tor) and two populations of *S. littorea* (Li-Car and Li-Pun). The populations of *S. psammitis* from Candeleda and Játar (Ps-Can, Ps-Jat) are not present in the nrDNA phylogenetic analyses because we obtained paralogous sequences during the assemblies (the average distance to the mean patristic distance within the ingroup was 87.5%). We tried to recover the orthologous copies by remapping the reads using the consensus sequence of *S. psammitis* from Benahavis (Ps-Ben) as a reference, but this failed to produce homologous sequences. The network analysis of the nrDNA data produced a set of relationships largely congruent with the phylogenetic results described above. There were four clusters of samples that were largely biogeographically aligned— "Northwest–West Iberian Peninsula," "South–Southwestern Iberian Peninsula," "Southeast Iberian Peninsula," and "Balearic Islands" (**Supplementary Figure S2C**).

### Gene Tree–Species Tree Reconciliation

**Figure 4:**
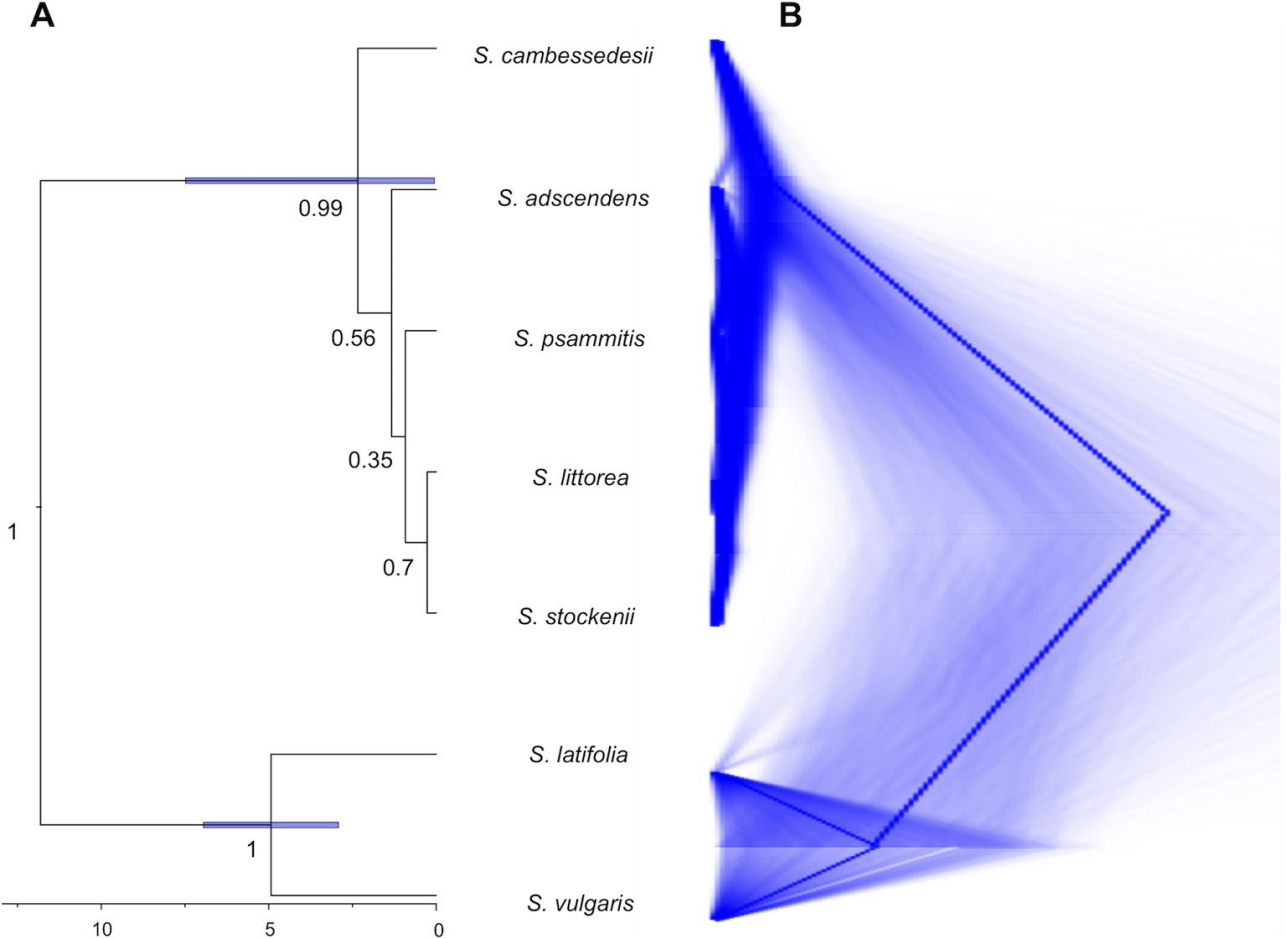
Species tree reconciliation analysis. **(A)** *BEAST multispecies coalescent with posterior probabilities over branches and node age estimates (95% HPD) with posterior probability limit set up to 0.90 (strong support). **(B)** DensiTree visualization of the posterior distribution of gene trees obtained from *BEAST.

For this and all subsequent analyses, we focus on the cpDNA genome because this organelle traces colonization events (although chloroplast capture could entangle phylogenetic relationships), which are particularly informative to clarify evolutionary relationships at the intra- and interspecific level, whereas nrDNA reflects both seed and pollen gene flows. The species tree obtained from *BEAST analysis of the cpDNA genome clustered together the five species of the *Silene* section *Psammophilae* (*PP* = 1; **Figure 4A**). The island populations of *S. cambessedesii* are strongly supported (*PP* = 0.99) as sister clade to the remaining mainland species, which showed weakly supported relationships among them. The species trees from DensiTree highlight the network-like relationships within the section, especially among mainland taxa (**Figure 4B**). The most recent common ancestor of the section emerged approximately 2.34 Ma (95% HPD: 0.05–7.48 Ma), while the origins of *S. adscendens*, *S. littorea*, *S. psammitis*, and *S. stockenii* are more recent and very similar to one another [∼1.33 (95% HPD: 0.02–4.13) Ma] (**Figure 4A**).

### Population Genetic Structure

**Figure 5:**
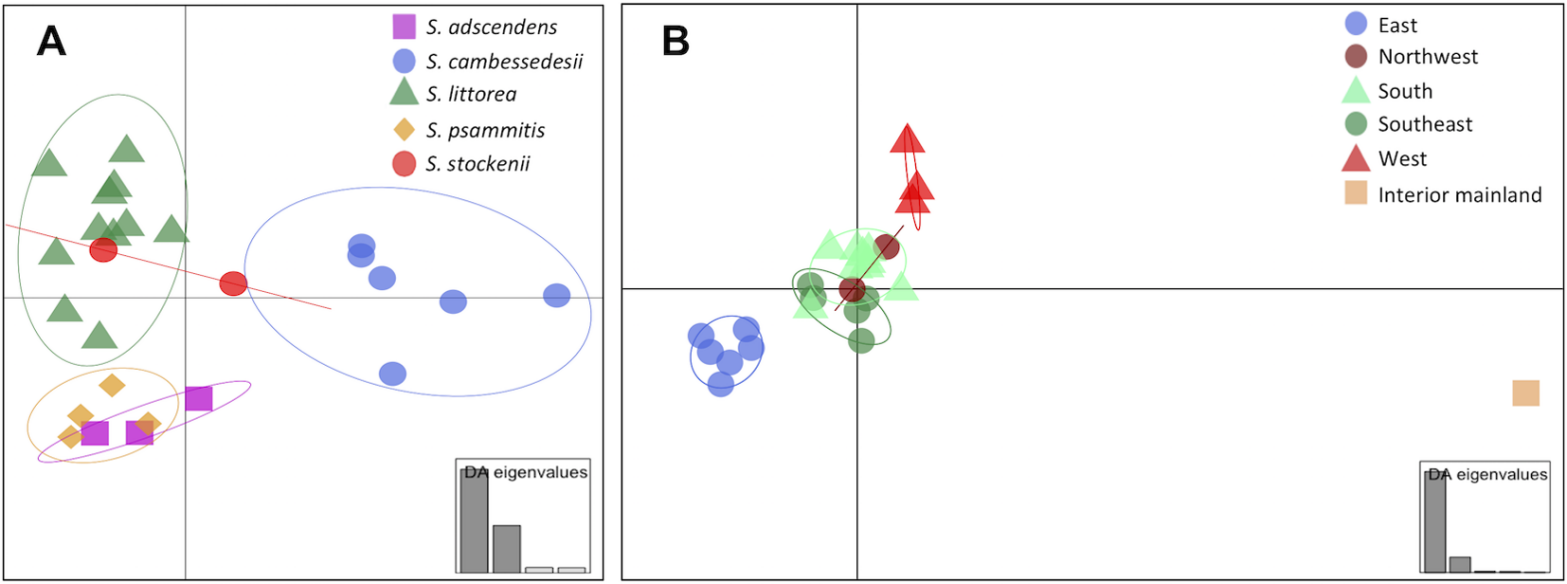
Scatter plot of Discriminant Analysis of Principal Components (DAPC) when using species and location categories as priors. In the scatter plot using species categories as priors **(A)**, each species is represented by different symbols and colors (*S. adscendens*, pink square; *S. cambessedesii*, blue circle; *S. littorea*, green triangle; *S. psammitis*, orange diamond; *S. stockenii*, red circle). In the scatter plot using geographic location categories as priors **(B)**, each location is represented by different symbols and colors (northwest, dark red circles; west, red triangles; south, green triangles; southeast, dark green circles; east, blue circles; interior mainland, brown square). The 95% inertial ellipses around each cluster represent the variance of the two first principal components of the DAPC. The insets represent the relative magnitude of the eigenvalues of the first four and five principal components, respectively.

We used DAPC of the plastid genome to investigate species affinities and to look for traces of organellar introgression. DAPC analysis revealed that the probability of membership to the assigned species priors was unequivocal for *S. cambessedesii* (100%), but variable for mainland populations, ranging from 12.7% to 100% (**Figure 2**). *S. cambessedesii* samples from the Balearic Islands were mostly separated from mainland populations along the first two retained principle component axes of the DAPC (representing 66.5% of the variation), whereas mainland populations showed largely overlapping 95% inertial ellipses (**Figure 5A**).

When using geography as priors, the probability of correct assignment to samples with regard to geographic location was again unequivocal for Balearic populations of *S. cambessedesii* (100%) and varied widely for the mainland populations (16.2–100%). *S. cambessedesii* and *S. psammitis* population from Candeleda (named as east and interior mainland locations, respectively, in **Figure 5B**) are clearly separated from the populations from the northwest, west, south, and southeast of the Iberian Peninsula, which overlapped along the first two retained principle component axes of the DAPC (**Figure 5B**).

The BAP analysis, performed to determine if the cpDNA data could be subdivided, conclusively found that there is just one genetic cluster. The log likelihood values for *K* *_1_* were much higher than those for other partitions (*K* *_1_* = −37,598.87, *K* *_2_* = −39,489.59, *K* *_3_* = −39,576.70, *K* *_4_* = −40,069.07).

Partial Mantel tests were performed to test for isolation-by-distance and showed that pairwise genetic distances were very low for island populations, but variable for mainland populations. Partial Mantel test showed spatial correlation of patristic distances when all populations were considered (*P* < 0.001, *r* = 0.35). However, when the partial Mantel tests were conducted separately for mainland and island populations, there was no significant correlation among mainland population (*P* = 0.27, *r* = 0.07), but there was marginally significant correlation among island populations (*P* = 0.079, *r* = 0.49) (**Supplementary Figure S3**).

We conducted AMOVA using the cpDNA data set in order to determine the distribution of genetic variation within and among species. There was a moderate level of genetic differentiation among species (*F* *_ST_* = 0.23; *P* < 0.001); most of the genetic variation was concentrated within species (77.3%) compared to among species (22.7%; **Table 2**). However, when the AMOVA was restricted to mainland species, 100% of the genetic variation lies among populations within species (**Table 3**). Finally, when the AMOVA was restricted to island populations, we did not find genetic differentiation between the two Balearic Islands (**Table 4**).

**Table 2:** Analysis of molecular variance (AMOVA) testing genetic subdivision among all species.

Source of variation	d.f.	Sum of squares	Variance components	Percentage of variation	Statistics	*P*
Among species	4	1,546.5	47.7	22.7	*F* *_ST_* = 0.23	<0.001
Within species	21	3,402.5	162.0	77.3		
Total	25	4,949.0	209.7			

**Table 3:** AMOVA testing genetic subdivision among mainland species.

Source of variation	d.f.	Sum of squares	Variance components	Percentage of variation	Statistics	*P*
Among species	3	552.9	−3.61	0	*F* *_ST_* = −0.018	0.58
Within species	16	3,189.4	199.3	100		
Total	19	3,742.3	195.7			

**Table 4:** AMOVA testing genetic subdivision between islands.

Source of variation	d.f.	Sum of squares	Variance components	Percentage of variation	Statistics	*P*
Among islands	1	42.2	−1.89	0	*F* *_ST_* = −0.04	0.71
Within islands	4	191.3	47.8	100		
Total	5	233.5	45.9			

### Similarity of *S. cambessedesii* Plants From Mainland and Balearic Populations

Sanger sequencing of four loci (*trnK* and *ycf1* from the cpDNA*, atp1* from the mtDNA, and ITS from nrDNA) from 20 seeds from a mainland population of *S. cambessedesii* (Almenara) generated 2,993 bp, which were compared to the Balearic Island populations sequenced using next-generation. Nearly all the sequences from the Almenara population were identical to those from the Balearic populations (99.2%). For the 23 SNPs (0.8%) that contained ambiguities in one or more of the Balearic populations, the Almenara individuals had one of the bases that causes the ambiguity. Two SNPs were exclusively found in the ITS1 and ITS2 regions of sequences from Balearic populations. SNPs detected in both island and mainland populations of *S. cambessedesii* were also present in several populations from the southeast and the southern end of the Iberian Peninsula. One SNP in the *ycf1* region was exclusively found in all *S. cambessedesii* samples.

In a ML analysis of the *trnK* fragment (cpDNA) including the mainland *S. cambessedesii* samples, only 3 of 28 (10.7%) internal branches were strongly supported (BS = 100) (**Supplementary Figure S4A**). The Almenara population showed a weak relationship with two populations of *S. cambessedesii* from the Balearic Islands, but also with three mainland populations from the south and the southeastern parts of the Iberian Peninsula. In the *ycf1*-based tree (cpDNA), 3 of 28 (10.7%) internal branches showed strong support (**Supplementary Figure S4B**). The Almenara population formed a cluster with all populations of *S. cambessedesii* from the Balearic Islands but was only moderately supported (BS = 62). Within this cluster, only the relationship between Almenara and one population from Ibiza (Ca-Cav) was strongly supported (BS = 97).

In the topology of the *atp1*-based tree (mtDNA), only 2 of 28 (7.1%) internal branches had a moderate or strong support (**Supplementary Figure S4C**). *S. cambessedesii* from Almenara and the Balearic Islands clustered together with three more mainland populations of other species, showing moderate support (BS = 75).

The ML analysis of the ITS region (nrDNA) revealed that 5 of 28 internal branches (17.9%) have moderate to strong support (**Supplementary Figure S4D**). All populations of *S. cambessedesii* from the Balearic Islands formed a very weakly supported clade (BS = 61), but plants from Almenara seemed to be more closely related with other mainland populations.

## Discussion

This study sought to reconstruct the relationships among the five species in *Silene* section *Psammophilae*, yet neither the complete chloroplast genome, nor the complete nrDNA cistron, nor a portion of the mitochondrial genome supported reciprocal monophyly of any of the species except the Balearic Island populations of *S. cambessedesii*. In the following sections, we discuss the potential causes for the incongruence among these three loci and between the DNA sequences and morphology-based species boundaries. Finally, we discuss the utility of genome skimming to obtain next-generation sequence data from three genomes for phylogeographic inference.

### Phylogenetic Relationships and Hybridization in Iberian *Silene*

DNA sequence data from all three genomes support a single common ancestor of *Silene* section *Psammophilae*, yet these data did not resolve the phylogenetic relationships therein. Most of the relationships across all three trees are poorly resolved, and a few results even indicate strongly supported, yet incongruent, relationships among the three genomes. Often, geography was a better predictor of relatedness than either morphology-based species boundaries or edaphic preferences of the species. The cpDNA analysis revealed a monophyletic clade formed by the six Balearic populations and another clade of three Cadiz populations representing two distinct species, whereas nrDNA analysis showed a clade formed by five Almeria populations representing two distinct species. Clearly, these three genomes reveal a complex evolutionary history of these species.

Incongruence among gene trees is frequently attributed to hybridization and/or incomplete lineage sorting (Frajman et al., 2009a). The lack of genetic differentiation found in the AMOVA analysis indicates that mainland species have not diverged genetically, probably because of a history of gene flow among them. Hybridization and introgression are common in *Silene* (Frajman and Oxelman, 2007; Frajman et al., 2009a; Rautenberg et al., 2010; Petri et al., 2013). Interspecific hybridization is often more common between closely related species (Mallet, 2005; Widmer et al., 2009), but in the genus *Silene*, hybridization has also been reported among distantly related species (Petri et al., 2013). The phylogenetic proximity and recent time of divergence (around 2.34 Ma) of species of section *Psammophilae* fall well within the possibility of interspecific crossability in this promiscuous genus, yet no one has reported the existence of interspecific hybrids. However, populations at the overlapping margins of the geographic distributions of distinct species have been noted to have intermediate morphological traits (EN personal observations).

In spite of the widespread incongruence between morphology-based species boundaries and phylogenetic relationships, we found occasional evidence of geographic patterning within Section *Psammophilae*. In addition to the monophyly of Balearic samples, in the southern end of the Iberian Peninsula (Cadiz), the proximity of one population of *S. stockenii* (St-Bar) to two populations of *S. littorea* (Li-Bre and Li-Tra), separated by only a few kilometers, may reflect an increased likelihood of gene flow. This could explain the largely overlapping ellipses defining these two species in the DAPC analysis. Similarly, in the nrDNA phylogenetic analysis, five geographically adjacent populations of *S. adscendens* and *S. littorea* from the southeastern part of the Iberian Peninsula cluster together (Ad-Feo, Ad-Ger, Ad-Tor, Li-Car, and Li-Pun). DAPC results show mixed genetic heritage in two of these populations (Ad-Feo and Li-Car) that contrasts with the other three genetically unique populations (Ad-Ger, Ad-Tor, and Li-Pun), which seem to become more isolated in the eastern part of the Baetic System. Our results suggest that, in some cases, geographic proximity of morphologically distinct species increases the probability of interspecific hybridization or introgression. Several studies have revealed that genetic diversity in *Silene* is more reflective of the geography than the taxonomy (e.g. Frajman et al., 2009a; Durović et al., 2017). In addition, genetic admixture between species highly differentiated for numerous phenotypic and ecological traits has been reported in some *Silene* species (e.g. *S. latifolia* and *S. dioica;* Minder et al., 2007; Hathaway et al., 2009), but also in many other genera (e.g. *Populus alba* and *Populus tremula*; Lexer et al., 2010). In the same way, even if the species of the Section *Psammophilae* display morphological variation among them and distinct edaphic affinities, these differences do not seem to preclude gene flow.

Alternatively, the lack of correlation between taxonomy and genetic structure in species of the Section *Psammophilae* could indicate that *Silene* section *Psammophilae* consists of a single species, or maybe two species if we consider that *S. cambessedesii* is genetically distinct from the mainland species. This would appear to be the most likely conclusion from the *BEAST analysis, which generates a largely unresolved tree (**Figure 4B**). Under this scenario, the existing geographically structured variation would have to be a product of genetic drift, phenotypic plasticity, and/or local adaptation. Due to the consistency of the morphological and ecological differences among these lineages, including important phenotypic traits for species identification in Caryophyllaceae such as floral morphology and seed-coat ornamentation (Chater and Walters, 1964; Talavera, 1990), we prefer to treat them as distinct species which we now know harbor cpDNA and portions of the mtDNA and nrDNA with evolutionary histories that are largely incongruent with the morphological boundaries. Additional markers spanning the nuclear genome (e.g. RAD-Seq, comparative transcriptomics, or whole genome sequencing) could be used to test for phylogenetic signal amidst a history of introgressive hybridization among species of the Section *Psammophilae*.

### Geographic Isolation Promotes Genetic Differentiation of Balearic Populations

The colonization of islands by one or a few individuals can lead to the fixation of genetic variation in contrast to large, contiguous continental populations (Frankham, 1997; Franks, 2010). The Balearic populations of *S. cambessedesii* may have fixed genetic differences due to founder effects following the colonization of these Mediterranean islands by a small number of individuals and/or subsequent population bottlenecks (Barton and Charlesworth, 1984; Carson and Templeton, 1984; Ellstrand and Elam, 1993; Orsini et al., 2013). The AMOVA analyses confirmed moderate genetic differentiation of this species with respect to mainland species of Section *Psammophilae*, but also showed the genetic uniformity of all Balearic populations. Genetic diversity in island populations may be influenced by numerous factors, but physical characteristics, such as the distance to other islands and the mainland, are probably one of the most important (García-Verdugo and Fay, 2014; Stuessy et al., 2014). For instance, proximity of California Channel Islands to each other and the mainland precludes island isolation as measured by the genetic diversity of endemic *Acmispon* (McGlaughlin et al., 2014). In contrast, the much larger geographical isolation of Hawaiian silverswords is an impediment to gene flow among islands and to any distant continents (McGlaughlin and Friar, 2011). Ibiza and Formentera are close enough to each other for dispersal to allow repeated gene flow between islands. Moreover, during the Pleistocene glaciations, Ibiza and Formentera formed a single large island as a consequence of sea level drop that allowed the contact among previously isolated populations of *S. cambessedesii* (Rodríguez et al., 2013; Chueca et al., 2015). Therefore, the weak genetic differentiation between islands we observe today is likely due to gene flow facilitated by historical land bridges or island proximity. By contrast, the islands remained isolated from the mainland during this time.

The presence of many endemic species in the Balearic Islands has been frequently explained by colonization events across land corridors that connected the archipelago to the Iberian Peninsula during the Messinian Salinity Crisis (∼5.5 Ma) (e.g. Garnatje et al., 2013; Chueca et al., 2015). Since the origin of Section *Psammophilae* seems to be more recent, the presence of *S. cambessedesii* in the Balearic Islands could not be explained by stepwise dispersal across these land corridors. Although *S. cambessedesii* lacks any obvious dispersal mechanisms, other species in this genus are capable of long-distance dispersal (Giles and Goudet, 1997; Eggens et al., 2007; Gussarova et al., 2015). Seed dispersal of coastal species without obvious dispersal mechanisms has been commonly explained by the accidental ingestion of seeds by granivorous birds, seed transportation in the plumage of birds and in mud attached to their legs and feet, or by water dispersal (Carlquist, 1967; Fridriksson, 1975; Richardson et al., 2000). We thus suggest that a single or very few long-distance dispersal events allowed *S. cambessedesii* to colonize the coasts of the Balearic Islands where they became isolated from the rampant interspecific hybridization on the Iberian Peninsula.

The existence of additional populations of *S. cambessedesii* on the mainland might be explained by dispersal from the islands back to the mainland following the genetic differentiation of this species on the Balearic Islands. The genetic diversity in many species distributed on both mainland and islands is highly influenced by gene flow between populations on both sides of water barriers (García-Verdugo et al., 2010). For instance, gene flow between populations of the Japanese shrub, *Weigela coraeensis,* from the Izu Peninsula and the adjacent northern Izu Islands explains why there is genetic differentiation with respect to more isolated populations from the southern islands (Yamada and Maki, 2012). Similarly, several studies have described an east–west geographical pattern in the Canary Islands in which populations from the eastern islands were genetically more similar to mainland taxa (e.g. García-Verdugo et al., 2009). *Silene hifacensis,* restricted to the east coast of the Iberian Peninsula and Ibiza, lacks any clear genetic differentiation between populations at both sides of the water barrier (Prentice et al., 2003). Gene flow between island and mainland populations of *S. cambessedesii* may explain the presence of a divergent nucleotide in the *ycf1* region restricted to this species. However, the remaining divergent SNPs found in *S. cambessedesii* sequences are also shared with several populations from the southeast and the southern end of the Iberian Peninsula. The larger effective population sizes of these mainland populations may continue to harbor genetic variation from before the dispersal event to the archipelago and/or have acquired variation more common in the mainland populations due to hybridization and introgression. Thus, our results suggest that genetic traces observed in DNA sequences of Almenara individuals are the result of at least one dispersal event from the islands back to the mainland, followed by introgression with mainland populations. Nevertheless, future detailed phylogeographic studies applying additional nuclear markers across as many mainland and island populations as possible will be necessary to further investigate the colonization history of *S. cambessedesii* in the Balearic Islands.

### On the Relative Accuracy of Reference-Guided Assembly of Each Genome

Reference-guided genome skimming for data from three genomes was sufficient to obtain the complete nuclear ribosomal unit and nearly complete plastome in all samples. The abundance of nrDNA and the high proportion of plastids per nuclear genome make them especially amenable for genome skimming and reference-guided assembly, even when there is no closely related genome to use as a reference (Straub et al., 2011; Straub et al., 2012). On the other hand, mitochondrial assemblies were largely incomplete and mainly restricted to coding regions, similar to other assemblies where mtDNAs were recovered (Malé et al., 2014; Ripma et al., 2014). Despite the abundance of this organelle in genomic DNA, obtaining mitochondrial genomes was challenging due to their complexity, variability, and frequent structural rearrangements in plants (Palmer and Herbon, 1988; Sugiyama et al., 2005; Knoop et al., 2011).

In some species of *Silene*, the mitochondrial genomes have a complex multichromosomal structure with large variations in genome sizes (Sloan et al., 2009), including the gain or loss of entire chromosomes in some species (Wu et al., 2015), and extremely variable substitution rates within and among species (Sloan et al., 2008; Sloan et al., 2009). In the same way, mitochondrial substitution rates also could vary among species of Section *Psammophilae*. In fact, the branch length of the *S. littorea* from Barra (Li-Bar) in the mtDNA-based tree is approximately 10 times longer than its sister taxon (*S. psammitis*) and other populations of the same species. If these differences in branch length are explained by an exceptional variation in mitochondrial substitution rates, and not because of an inaccurate assembly, caution would be needed when using mitochondrial sequence for reconstructing phylogenetic relationships (Felsenstein, 1978). Larger fragments of the mitochondrial genome, including introns and intergenic regions, will be necessary to further examine phylogenetic and biogeographic analyses using this genome (Straub et al., 2012; Bock et al., 2014). Thus, genome skimming is an efficient approach to generate the majority of the chloroplast genome, nrDNA cistron, and some mitochondrial coding sequences, yet even this cannot overcome the complex, recent, reticulate evolutionary history of *Silene* section *Psammophilae*.

## Conclusions

In this study, we skimmed the chloroplast genome, complete nrDNA, and portions of the mitochondrial genome, yet this was largely insufficient to reconstruct the complex evolutionary history of the members of *Silene* section *Psammophilae*. Except in the presence of substantial biogeographic barriers (e.g. the Balearic Islands), the highly reticulated evolutionary histories of young lineages and pervasive hybridization will remain challenging, even with massive amounts of sequence data at hand.

## Data Availability Statement

The datasets generated for this study can be found in the Genbank (MN365968 - MN365993, MN334914 - MN334939, MN334784 - MN334809, MN334810 - MN334835, MN334836 - MN334861, MN334862 - MN334887, MN334888 - MN334913, MN325944 - MN325960).

## Author Contributions

EN, MB, and JW conceived of the experiments. MB, IC-S, and EN carried out the sampling. IC-S and JW performed the DNA extraction. JW and JV performed the assembly and the alignment of sequences. JW and JV performed the Sanger sequencing for SNP validation. Phylogenetic analyses were conducted by JV and JW. JV and JW wrote the article, with assistance from all coauthors. All authors read and approved the final manuscript.

## Funding

This work was supported with FEDER funds by the Spanish Government MINECO projects (CGL2009-08257, CGL2012-37646, and CGL2015-63827-P) and the Predoctoral Training Program grants to IS (BES-2010-031073) and JV (BES-2013–062610).

## Conflict of Interest

The authors declare that the research was conducted in the absence of any commercial or financial relationships that could be construed as a potential conflict of interest.

## References

[B1] Arnold M. L. (1997).Natural hybridization and evolution. Oxford, U.K: Oxford University Press.

[B2] Baker H. G. (1948). Stages in invasion and replacement demonstrated by species of *Melandrium*. J. Ecol. 36, 96–119. 10.2307/2256649

[B3] Balounova V. Gogela R. Cegan R. Cangren P. Zluvova J. Safar J. (2019). Evolution of sex determination and heterogamety changes in section *Otites* of the genus *Silene*. Sci. Rep. 9, 1045. 10.1038/s41598-018-37412-x 30705300 PMC6355844

[B4] Barr C. M. Keller S. R. Ingvarsson P. K. Sloan D. B. Taylor D. R. (2007). Variation in mutation rate and polymorphism among mitochondrial genes of *Silene vulgaris*. Mol. Biol. Evol. 24, 1783–1791. 10.1093/molbev/msm106 17533174

[B5] Barton N. H. Charlesworth B. (1984). Genetic revolutions, founder effects, and speciation. Annu. Rev. Ecol. Syst. 15, 133–164. 10.1146/annurev.es.15.110184.001025

[B6] Bernasconi G. Antonovics J. Biere A. Charlesworth D. Delph L. F. Filatov D. (2009). *Silene* as a model system in ecology and evolution. Heredity (Edinb). 103, 5–14. 10.1038/hdy.2009.34 19367316

[B7] Bittkau C. Comes H. P. (2009). Molecular inference of a Late Pleistocene diversification shift in *Nigella* s. lat. (Ranunculaceae) resulting from increased speciation in the Aegean archipelago. J. Biogeogr. 36, 1346–1360. 10.1111/j.1365-2699.2008.02003.x

[B8] Bock D. G. Kane N. C. Ebert D. P., and ,L. H. (2014). Genome skimming reveals the origin of the Jerusalem Artichoke tuber crop species: neither from Jerusalem nor an artichoke. New Phytol. 201, 1021–1030. 10.1111/nph.12560 24245977

[B9] Bouckaert R. Heled J. (2014). DensiTree 2: seeing trees through the forest. bioRxiv 10, 012401. 10.1101/012401

[B10] Bouckaert R. Heled J. Kühnert D. Vaughan T. Wu C. H. Xie D. (2014). BEAST 2: a software platform for Bayesian evolutionary analysis. PloS Comput. Biol. 10, e1003537. 10.1371/journal.pcbi.1003537 24722319 PMC3985171

[B11] Carlquist S. (1967). The biota of long-distance dispersal. V. Plant dispersal to Pacific Islands. Bull. Torrey Bot. Club 94, 129–162. 10.2307/2484044

[B12] Carson H. L. Templeton A. R. (1984). Genetic revolutions in relation to speciation phenomena: the founding of new populations. Annu. Rev. Ecol. Syst. 15, 97–131. 10.1146/annurev.es.15.110184.000525

[B13] Casimiro-Soriguer I. (2015). Sistemas sexuales y polimorfismo de color en *Silene*: una aproximación en la sección *Psammophilae*. Ph.D. Dissertation. Seville: Pablo de Olavide University.

[B14] Chater O. A. Walters S. M. (1964). Silene. Flora Europaea, Vol. 1 . Eds. Tutin T. G. Heywood V. H. Burges N. A. Valentine D. H. Walters S. M. (Cambridge, UK: Cambridge University Press), 158–181.

[B15] Chueca L. J. Madeira M. J. Gómez-Moliner B. J. (2015). Biogeography of the land snail genus *Allognathus* (Helicidae): middle Miocene colonization of the Balearic Islands. J. Biogeogr. 42, 1845–1857. 10.1111/jbi.12549

[B16] Corander J. Marttinen P. Sirén J. Tang J. (2008). Enhanced Bayesian modelling in BAPS software for learning genetic structures of populations. BMC Bioinf. 9, 539. 10.1186/1471-2105-9-539 PMC2629778 19087322

[B17] Darriba D. Taboada G. L. Doallo R. Posada D. (2012). jModelTest 2: more models, new heuristics and high-performance computing. Nat. Methods 9, 772. 10.1038/nmeth.2109 PMC4594756 22847109

[B18] Dick C. A. Buenrostro J. Butler T. Carlson M. L. Kliebenstein D. J. Whittall J. B. (2011). Arctic mustard flower color polymorphism controlled by petal-specific downregulation at the threshold of the anthocyanin biosynthetic pathway. PloS One 6, e18230. 10.1371/journal.pone.0018230 21490971 PMC3072389

[B19] Drummond A. J. Suchard M. A. Xie D. Rambaut A. (2012). Bayesian phylogenetics with BEAUti and the BEAST 1.7. Mol. Biol. Evol. 29, 1969–1973. 10.1093/molbev/mss075 22367748 PMC3408070

[B20] Du Pasquier P. E. Jeanmonod D. Naciri Y. (2017). Morphological convergence in the recently diversified *Silene gigantea* complex (Caryophyllaceae) in the Balkan Peninsula and south-western Turkey, with the description of a new subspecies. Bot. J. Linn. Soc 183, 474–493. 10.1093/botlinnean/bow016

[B21] Duggen S. Hoernle K. Bogaard P. Van Den R. L. Morgan J. P. (2003). Deep roots of the Messinian salinity crisis. Nat. 422, 602–606. 10.1038/nature01551.1 12686997

[B22] Durović S. Schönswetter P. Niketić M. Tomović G. Frajman B. (2017). Disentangling relationships among the members of the Silene saxifraga alliance (Caryophyllaceae): Phylogenetic structure is geographically rather than taxonomically segregated. Taxon 66, 343–364. 10.12705/662.4

[B23] Eggens F. Popp M. Nepokroeff M. Wagner W. L. Oxelman B. (2007). The origin and number of introductions of the Hawaiian endemic *Silene* species (Caryophyllaceae). Am. J. Bot. 94, 210–218. 10.3732/ajb.94.2.210 21642223

[B24] Ellstrand N. C. Elam D. R. (1993). Population genetic consequences of small population size: implications for plant conservation. Annu. Rev. Ecol. Syst. 24, 217–242. 10.1146/annurev.es.24.110193.001245

[B25] Erixon P. Oxelman B. (2008). Reticulate or tree-like chloroplast DNA evolution in *Sileneae* (Caryophyllaceae)?. Mol. Phylogenet. Evol. 48, 313–325. 10.1016/j.ympev.2008.04.015 18490181

[B26] Excoffier L. Lischer H. E. L. (2010). Arlequin suite ver 3.5: a new series of programs to perform population genetics analyses under Linux and Windows. Mol. Ecol. Resour. 10, 564–567. 10.1111/j.1755-0998.2010.02847.x 21565059

[B27] Excoffier L. Smouse P. E. Quattro J. M. (1992). Analysis of molecular variance inferred from metric distances among DNA haplotypes: application to human mitochondrial DNA restriction data. Genet. 131, 479–491. 10.1093/genetics/131.2.479 PMC1205020 1644282

[B28] Felsenstein J. (1978). Cases in which parsimony and compatibility methods will be positively misleading. Syst. Zool. 27, 401–410. 10.1093/sysbio/27.4.401

[B29] Frajman B. Eggens F. Oxelman B. (2009a). Hybrid origins and homoploid reticulate evolution within *Heliosperma* (*Sileneae*, Caryophyllaceae) - A multigene phylogenetic approach with relative dating. Syst. Biol. 58, 328–345. 10.1093/sysbio/syp030 20525587

[B30] Frajman B. Heidari N. Oxelman B. (2009b). Phylogenetic relationships of Atocion and Viscaria (*Sileneae*, Caryophyllaceae) inferred from chloroplast, nuclear ribosomal, and low-copy gene DNA sequences. Taxon 58, 811–824. 10.1002/tax.583010

[B31] Frajman B. Oxelman B. (2007). Reticulate phylogenetics and phytogeographical structure of *Heliosperma* (*Sileneae*, Caryophyllaceae) inferred from chloroplast and nuclear DNA sequences. Mol. Phylogenet. Evol. 43, 140–155. 10.1016/j.ympev.2006.11.003 17188521

[B32] Frankham R. (1997). Do island populations have less genetic variation than mainland populations?. Heredity (Edinb). 78, 311–327. 10.1038/hdy.1997.46 9119706

[B33] Franks S. J. (2010). Genetics, evolution, and conservation of Island plants. J. Plant Biol. 53, 1–9. 10.1007/s12374-009-9086-y

[B34] Fridriksson S. (1975).Surtsey, Evolution of Life on a Volcanic Island. Butterworth London, UK: John Wiley.

[B35] García-Verdugo C. Fay M. F. (2014). Ecology and evolution on oceanic islands: broadening the botanical perspective. Bot. J. Linn. Soc 174, 271–275. 10.1111/boj.12154

[B36] García-Verdugo C. Fay M. F. Granado-Yela C. De Casas R. R. Balaguer L. Besnard G. (2009). Genetic diversity and differentiation processes in the ploidy series of *Olea europaea* L.: a multiscale approach from subspecies to insular populations. Mol. Ecol. 18, 454–467. 10.1111/j.1365-294X.2008.04027.x 19143937

[B37] García-Verdugo C. Forrest A. D. Fay M. F. Vargas P. (2010). The relevance of gene flow in metapopulation dynamics of an oceanic island endemic, *Olea Europaea* subsp. *Guanchica* . Evol. (N. Y). 64, 3525–3536. 10.1111/j.1558-5646.2010.01091.x 20666841

[B38] Garnatje T. Pérez-Collazos E. Pellicer J. Catalán P. (2013). Balearic insular isolation and large continental spread framed the phylogeography of the western Mediterranean *Cheirolophus intybaceus s.l.* (Asteraceae). Plant Biol. 15, 166–175. 10.1111/j.1438-8677.2012.00632.x 22759527

[B39] Giles B. E. Goudet J. (1997). Genetic differentiation in *Silene dioica* metapopulations: estimation of spatiotemporal effects in a successional plant species. Am. Nat. 149, 507–526. 10.1086/286002

[B40] Greuter W. (1995). *Silene* (Caryophyllaceae) in Greece: a subgeneric and sectional classification. Taxon 44, 543–581. 10.2307/1223499

[B41] Gussarova G. Allen G. A. Mikhaylova Y. McCormick L. J. Mirré V. Marr K. L. (2015). Vicariance, long-distance dispersal, and regional extinction-recolonization dynamics explain the disjunct circumpolar distribution of the arctic-alpine plant *Silene acaulis*. Am. J. Bot. 102, 1703–1720. 10.3732/ajb.1500072 26437887

[B42] Hathaway L. Malm J. U. Prentice H. C. (2009). Geographically congruent large-scale patterns of plastid haplotype variation in the European herbs Silene dioica and S. latifolia (Caryophyllaceae). Biol. J. Linn. Soc 161, 153–170. 10.1111/j.1095-8339.2009.01003.x

[B43] Heled J. Drummond A. J. (2010). Bayesian Inference of Species Trees from Multilocus Data using *BEAST. Mol. Biol. Evol. 27, 570–580. 10.1093/molbev/msp274 19906793 PMC2822290

[B44] Holland B. R. Benthin S. Lockhart P. J. Moulton V. Huber K. T. (2008). Using supernetworks to distinguish hybridization from lineage-sorting. BMC Evol. Biol. 8, 202. 10.1186/1471-2148-8-202 18625077 PMC2500029

[B45] Hsü K. J. Ryan W. B. Cita M. B. (1973). Late Miocene desiccation of the Mediterranean. Nat. 242, 240–244. 10.1038/242240a0

[B46] Huelsenbeck J. P. Ronquist F. (2001). MRBAYES: Bayesian inference ofphylogenetic trees. Bioinforma. Appl. Note 17, 754–755. 10.1093/bioinformatics/17.8.754 11524383

[B47] Huson D. H. Bryant D. (2006). Application of phylogenetic networks in evolutionary studies. Mol. Biol. Evol. 23, 254–267. 10.1093/molbev/msj030 16221896

[B48] Joly S. McLenachan P. A. Lockhart P. J. (2009). A statistical approach for distinguishing hybridization and incomplete lineage sorting. Am. Nat. 174, E54–E70. 10.1086/600082 19519219

[B49] Jombart T. (2008). *adegenet*: a R package for the multivariate analysis of genetic markers. Bioinf. 24, 1403–1405. 10.1093/bioinformatics/btn129 18397895

[B50] Jombart T. Collins C. A. (2015). Tutorial for Discriminant Analysis of Principal Components (DAPC) using adegenet 2.0.0. Available at: http://adegenet.r-forge.r-project.org/files/tutorial-dapc.pdf.

[B51] Jombart T. Devillard S. Balloux F. (2010). Discriminant analysis of principal components: anew method for the analysis of genetically structured populations. BMC Genet. 11, 94. 10.1186/1471-2156-11-94 20950446 PMC2973851

[B52] Juan A. Crespo M. B. Cowan R. S. Lexer C. Fay M. F. (2004). Patterns of variability and gene flow in *Medicago citrina*, an endangered endemic of islands in the western Mediterranean, as revealed by amplified fragment length polymorphism (AFLP). Mol. Ecol. 13, 2679–2690. 10.1111/j.1365-294X.2004.02289.x 15315680

[B53] Kane N. Sveinsson S. Dempewolf H. Yang J. Y. Zhang D. Engels J. M. M. (2012). Ultra-barcoding in cacao (*Theobroma* spp.; Malvaceae) using whole chloroplast genomes and nuclear ribosomal DNA. Am. J. Bot. 99, 320–329. 10.3732/ajb.1100570 22301895

[B54] Katoh K. Standley D. M. (2013). MAFFT multiple sequence alignment software version 7: improvements in performance and usability. Mol. Biol. Evol. 30, 772–780. 10.1093/molbev/mst010 23329690 PMC3603318

[B55] Kearse M. Sturrock S. Meintjes P. (2012). The Geneious 6.0.3 read mapper. Available at: https://assets.geneious.com/documentation/geneious/GeneiousReadMapper.pdf

[B56] Knoop V. Volkmar U. Hecht J. Grewe F. (2011). “Mitochondrial genome evolution in the plant lineage,” in Plant mitochondria (New York, NY: Springer), 3–29. 10.1007/978-0-387-89781-3_1

[B57] Krijgsman W. Hilgen F. J. Raffi I. Sierro F. J. Wilson D. S. (1999). Chronology, causes and progression of the Messinian salinity crisis. Nat. 400, 652–655. 10.1038/23231

[B58] Lexer C. Joseph J. A. Van Loo M. Barbará T. Heinze B. Bartha D. (2010). Genomic admixture analysis in European *Populus* spp. reveals unexpected patterns of reproductive isolation and mating. Genet. 186, 699–712. 101534/genetics.110.118828 10.1534/genetics.110.118828 PMC2954470 20679517

[B59] Malé P. J. G. Bardon L. Besnard G. Coissac E. Delsuc F. Engel J. (2014). Genome skimming by shotgun sequencing helps resolve the phylogeny of a pantropical tree family. Mol. Ecol. Resour. 14, 966–975. 10.1111/1755-0998.12246 24606032

[B60] Mallet J. (2005). Hybridization as an invasion of the genome. Trends Ecol. Evol. 20, 229–237. 10.1016/j.tree.2005.02.010 16701374

[B61] Mantel N. (1967). The detection of disease clustering and a generalized regression approach. Cancer Res. 27, 209–220. 6018555

[B62] McGlaughlin M. E. Friar E. A. (2011). Evolutionary diversification and geographical isolation in *Dubautia laxa* (Asteraceae), a widespread member of the Hawaiian silversword alliance. Ann. Bot. 107, 357–370. 10.1093/aob/mcq252 21193480 PMC3043929

[B63] McGlaughlin M. E. Wallace L. E. Wheeler G. L. Bresowar G. Riley L. Britten N. R. (2014). Do the island biogeography predictions of MacArthur and Wilson hold when examining genetic diversity on the near mainland California Channel Islands ? Examples from endemic *Acmispon* (Fabaceae). Bot. J. Linn. Soc 174, 289–304. 10.1111/boj.12122

[B64] Médail F. Quézel P. (1997). Hot-spots analysis for conservation of plant biodiversity in the Mediterranean Basin. Ann. Missouri Bot. Gard. 84, 112–127. 10.2307/2399957

[B65] Médail F. Quézel P. (1999). Biodiversity hotspots in the Mediterranean Basin: setting global conservation priorities. Conserv. Biol. 13, 1510–1513. 10.1046/j.1523-1739.1999.98467.x

[B66] Minder A. M. Rothenbuehler C. Widmer A. (2007). Genetic structure of hybrid zones between *Silene latifolia* and *Silene dioica* (Caryophyllaceae): evidence for introgressive hybridization. Mol. Ecol. 16, 2504–2516. 10.1111/j.1365-294X.2007.03292.x 17561909

[B67] Myers N. Mittermeier R. A. Mittermeier C. G. Da Fonseca G. A. B. Kent J. (2000). Biodiversity hotspots for conservation priorities. Nat. 403, 853–858. 10.1038/35002501 10706275

[B68] Naciri Y. Du Pasquier P. E. Lundberg M. Jeanmonod D. Oxelman B. (2017). A phylogenetic circumscription of *Silene* sect. *Siphonomorpha* (Caryophyllaceae) in the Mediterranean Basin. Taxon 66, 91–108. 10.12705/661.5

[B69] Navarro A. Ferrer-Gallego P. P. Ferrando I. Albert F. J. Martínez V. Escribá M. C. (2015). Experiencias de conservación activa e *in situ* con *Silene cambessedesii*, especie en peligro de extinción en la Comunidad Valenciana. Conserv. Veg. 19, 11–13.

[B70] Nieto Feliner G. (2014). Patterns and processes in plant phylogeography in the Mediterranean Basin. A review. Perspect. Plant Ecol. Evol. Syst. 16, 265–278. 10.1016/j.ppees.2014.07.002

[B71] Oksanen J. Blanchet F. Friendly M. Kindt R. Legendre P. McGlinn D. (2018). vegan: community ecology package. R package version 2.5-2. https://CRAN.R-project.org/package=vegan.

[B72] Okuyama Y. Fujii N. Wakabayashi M. Kawakita A. Ito M. Watanabe M. (2005). Nonuniform concerted evolution and chloroplast capture: heterogeneity of observed introgression patterns in three molecular data partition phylogenies of Asian *Mitella* (Saxifragaceae). Mol. Biol. Evol. 22, 285–296. 10.1093/molbev/msi016 15483320

[B73] Orsini L. Vanoverbeke J. Swillen I. Mergeay J. De Meester L. (2013). Drivers of population genetic differentiation in the wild: Isolation by dispersal limitation, isolation by adaptation and isolation by colonization. Mol. Ecol. 22, 5983–5999. 10.1111/mec.12561 24128305

[B74] Oxelman B. Lidén M. (1995). Generic boundaries in the tribe *Sileneae* (Caryophyllaceae) as inferred from nuclear rDNA sequences. Taxon 44, 525–542. 10.2307/1223498

[B75] Oxelman B. Liden M. Berglund D. (1997). Chloroplast *rps*l6 intron phylogeny of the tribe *Sileneae* (Caryophyllaceae). Plant Syst. Evol. 206, 393–410. 10.1007/BF00987959

[B76] Oxelman B. Rautenberg A. Thollesson M. Larsson A. Frajman B. Eggens F. (2013). Sileneae taxonomy and systematics. Available at: http://www.sileneae.info.

[B77] Oxelman B. Universitet U. Rabeler R. K. (2001). A revised generic classification of the tribe *Sileneae* (Caryophyllaceae). Nord. J. Bot. 20, 743–748. 10.1111/j.1756-1051.2000.tb00760.x

[B78] Palmer J. D. Herbon L. A. (1988). Plant mitochondrial DNA evolved rapidly in structure, but slowly in sequence. J. Mol. Evol. 28, 87–97. 10.1007/BF02143500 3148746

[B79] Parks M. Cronn R. Liston A. (2009). Increasing phylogenetic resolution at low taxonomic levels using massively parallel sequencing of chloroplast genomes. BMC Biol. 7, 84. 10.1186/1741-7007-7-84 19954512 PMC2793254

[B80] Petri A. Oxelman B. (2011). Phylogenetic relationship within *Silene* (Caryophyllaceae) section *Physolychnis*. Taxon 60, 953–968. 10.1002/tax.604002

[B81] Petri A. Pfeil B. E. Oxelman B. (2013). Introgressive hybridization between anciently diverged lineages of *Silene* (Caryophyllaceae). PloS One 8, e67729. 10.1371/journal.pone.0067729 23861793 PMC3704521

[B82] Popp M. Oxelman B. (2004). Evolution of a RNA polymerase gene family in *Silene* (Caryophyllaceae) - Incomplete concerted evolution and topological congruence among paralogues. Syst. Biol. 53, 914–932. 10.1080/10635150490888840 15764560

[B83] Popp M. Oxelman B. (2007). Origin and evolution of North American polyploid Silene (Caryophyllaceae). Am. J. Bot. 94, 330–349. 10.3732/ajb.94.3.330 21636405

[B84] Prentice H. C. Malm J. U. Mateu-Andres I. Segarra-Moragues J. G. (2003). Allozyme and chloroplast DNA variation in island and mainland populations of the rare Spanish endemic, *Silene hifacensis* (Caryophyllaceae). Conserv. Genet. 4, 543–555. 10.1023/A:1025603328704

[B85] R Core Team (2016). R: a language and environment for statistical computing. Available at: http://www.r-project.org.

[B86] Rambaut A. FigTree v1.4.2: tree figure drawing tool, 2014, Available at: http://tree.bio.ed.ac.uk/software/figtree.

[B87] Rambaut A. Suchard M. A. Xie D. Drummond A. Tracer v1.6, 2014, Available at: http://beast.bio.ed.ac.uk/Tracer.

[B88] Rautenberg A. Hathaway L. Oxelman B. Prentice H. C. (2010). Geographic and phylogenetic patterns in *Silene* section *Melandrium* (Caryophyllaceae) as inferred from chloroplast and nuclear DNA sequences. Mol. Phylogenet. Evol. 57, 978–991. 10.1016/j.ympev.2010.08.003 20723610

[B89] Rautenberg A. Sloan D. B. Aldén V. Oxelman B. (2012). Phenolic relationships of *Silene multinervia* and *Silene* section *Conoimorpha* (Caryophyllaceae). Syst. Bot. 37, 226–237. 10.1600/036364412X616792

[B90] Reyment R. A. (1983). Palaeontological aspects of island biogeography: colonization and evolution of mammals on Mediterranean islands. Oikos 41, 299–306. 10.2307/3544089

[B91] Richardson D. M. Allsopp N. Antonio C. M. D. Milton S. J. Rejma M. (2000). Plant invasions – the role of mutualisms. Biol. Rev. 75, 65–93. 10.1111/j.1469-185X.1999.tb00041.x 10740893

[B92] Rieseberg L. H. (1997). Hybrid origins of plant species. Annu. Rev. Ecol. Syst. 28, 359–389. 10.1146/annurev.ecolsys.28.1.359

[B93] Rieseberg L. H. Raymond O. Rosenthal D. M. Lai Z. Livingstone K. Nakazato T. (2003). Major ecological transitions in wild sunflowers facilitated by hybridization. Sci. 301, 1211–1216. 10.1126/science.1086949 12907807

[B94] Ripma L. A. Simpson M. G. Hasenstab-Lehman K. (2014). Geneious! Simplified genome skimming methods for phylogenetic systematic studies: a case study in *Oreocarya* (Boraginaceae). Appl. Plant Sci. 2, 1400062. 10.3732/apps.1400062 PMC4259456 25506521

[B95] Rodríguez V. Brown R. P. Terrasa B. Pérez-Mellado V. Castro J. A. Picornell A. (2013). Multilocus genetic diversity and historical biogeography of the endemic wall lizard from Ibiza and Formentera, *Podarcis pityusensis* (Squamata: Lacertidae). Mol. Ecol. 22, 4829–4841. 10.1111/mec.12443 23962158

[B96] Ruhsam M. Rai H. S. Mathews S. Ross T. G. Graham S. W. Raubeson L. A. (2015). Does complete plastid genome sequencing improve species discrimination and phylogenetic resolution in *Araucaria*?. Mol. Ecol. Resour. 15, 1067–1078. 10.1111/1755-0998.12375 25611173

[B97] Sloan D. B. Barr C. M. Olson M. S. Keller S. R. Taylor D. R. (2008). Evolutionary rate variation at multiple levels of biological organization in plant mitochondrial DNA. Mol. Biol. Evol. 25, 243–246. 10.1093/molbev/msm266 18056075

[B98] Sloan D. B. Oxelman B. Rautenberg A. Taylor D. R. (2009). Phylogenetic analysis of mitochondrial substitution rate variation in the angiosperm tribe *Sileneae*. BMC Evol. Biol. 9, 260. 10.1186/1471-2148-9-260 19878576 PMC2777880

[B99] Soltis D. E. Kuzoff R. K. (1995). Discordance between nuclear and chloroplast phylogenies in the *Heuchera* group (Saxifragaceae). Evol. (N. Y). 49, 727–742. 10.2307/2410326 28565145

[B100] Stamatakis A. (2014). RAxML version 8: a tool for phylogenetic analysis and post-analysis of large phylogenies. Bioinf. 30, 1312–1313. 10.1093/bioinformatics/btu033 PMC3998144 24451623

[B101] Straub S. C. K. Fishbein M. Livshultz T. Foster Z. Parks M. Weitemier K. (2011). Building a model: developing genomic resources for common milkweed (*Asclepias syriaca*) with low coverage genome sequencing. BMC Genomics 12, 211. 10.1186/1471-2164-12-211 21542930 PMC3116503

[B102] Straub S. C. K. Parks M. Weitemier K. Fishbein M. Cronn R. C. Liston A. (2012). Navigating the tip of the genomic iceberg: Next-Generation Sequencing for plant systematics. Am. J. Bot. 99, 349–364. 10.3732/ajb.1100335 22174336

[B103] Stuessy T. F. Takayama K. López-Sepúlveda P. Crawford D. J. (2014). Interpretation of patterns of genetic variation in endemic plant species of oceanic islands. Bot. J. Linn. Soc 174, 276–288. 10.1111/boj.12088 26074627 PMC4459035

[B104] Sugiyama Y. Watase Y. Nagase M. Makita N. Yagura S. Hirai A. (2005). The complete nucleotide sequence and multipartite organization of the tobacco mitochondrial genome: comparative analysis of mitochondrial genomes in higher plants. Mol. Genet. Genomics 272, 603–615. 10.1007/s00438-004-1075-8 15583938

[B105] Talavera S. (1979). Revisión de la sect. *Erectorefractae* Chowdhuri del género *Silene* L. Lagascalia 8, 135–164.

[B106] Talavera S. (1990). Silene in Flora Ibérica,Vol. 2 . Eds. Castroviejo S. Aedo C. Laínz M. Muñoz Garmendia F. Nieto Feliner G. Paiva J. (Madrid, Spain: Real Jardín Botánico) 313–406.

[B107] Thompson J. D. (2005).Plant evolution in the Mediterranean. New York, NY: Oxford University Press. 10.1093/acprof:oso/9780198515340.001.0001

[B108] Van der Geer A. Lyras G. de Vox J. Dermitzakis M. (2010).Evolution of island mammals: adaptation and extinction of placental mammals on islands. Oxford, U.K: Wiley-Blackwell.

[B109] Wang Y. Chen Q. Chen T. Tang H. Liu L. Wang X. (2016). Phylogenetic insights into Chinese *Rubus* (Rosaceae) from multiple chloroplast and nuclear DNAs. Front. Plant Sci. 7, 968. 10.3389/fpls.2016.00968 27446191 PMC4925702

[B110] Whittall J. B. Carlson M. L. Beardsley P. M. Meinke R. J. Liston A. (2006). The Mimulus moschatus alliance (Phrymaceae): molecular and morphological phylogenetics and their conservation implications. Syst. Bot. 31, 380–397. 10.1600/036364406777585810

[B111] Whittall J. B. Syring J. Parks M. Buenrostro J. Dick C. Liston A. (2010). Finding a (pine) needle in a haystack: chloroplast genome sequence divergence in rare and widespread pines. Mol. Ecol. 19, 100–114. 10.1111/j.1365-294X.2009.04474.x 20331774

[B112] Widmer A. Lexer C. Cozzolino S. (2009). Evolution of reproductive isolation in plants. Heredity (Edinb). 102, 31–38. 10.1038/hdy.2008.69 18648386

[B113] Wright S. (1951). The genetical structure of populations. Ann. Eugen. 15, 323–354. 10.1111/j.1469-1809.1949.tb02451.x 24540312

[B114] Wu Z. Cuthbert J. M. Taylor D. R. Sloan D. B. (2015). The massive mitochondrial genome of the angiosperm *Silene noctiflora* is evolving by gain or loss of entire chromosomes. Proc. Natl. Acad. Sci. 112, 10185–10191. 10.1073/pnas.1421397112 25944937 PMC4547255

[B115] Yamada T. Maki M. (2012). Impact of geographical isolation on genetic differentiation in insular and mainland populations of *Weigela coraeensis* (Caprifoliaceae) on Honshu and the Izu Islands. J. Biogeogr. 39, 901–917. 10.1111/j.1365-2699.2011.02634.x

