# Supplementary material for: Whole Plastome Sequencing Within Silene Section Psammophilae Reveals Mainland Hybridization and Divergence With the Balearic Island Populations
Source: Front Plant Sci. 2019 Nov 15;10:1466. doi: 10.3389/fpls.2019.01466 (PMC6872646; doi:10.3389/fpls.2019.01466)
Supplement: Supplementary file 1 [file DataSheet_1.pdf]

Supporting Information

Additional Supporting Information may be found in the online version of this article:

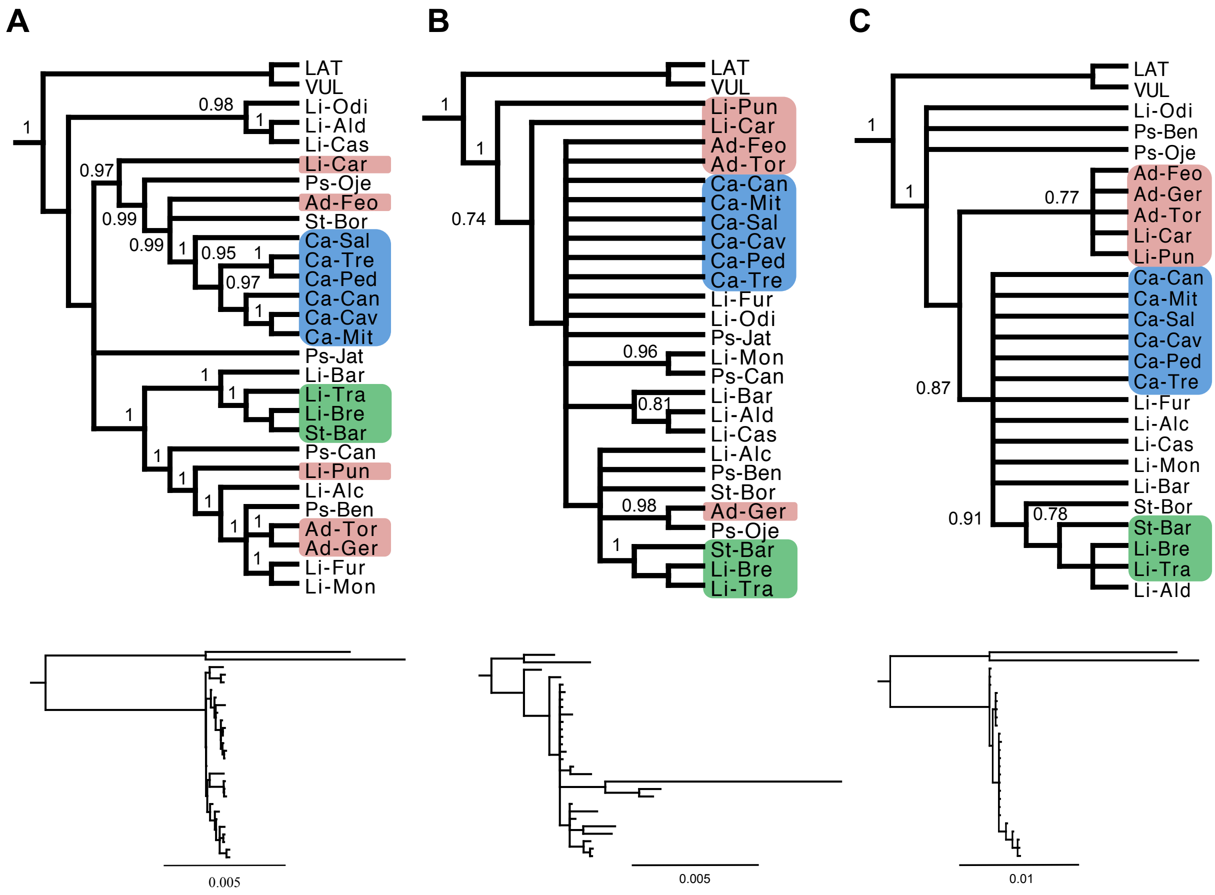

Figure S1: Phylogenetic relationships within the Section *Psammophilae* using Bayesian estimation. Bayesian trees from complete chloroplast genomes (A), six coding regions of the mitochondrial genome (B), and complete nuclear ribosomal cistron with a partial ETS (C). We applied the GTR+I+G model of sequence evolution for two separate runs, each consisting of four independent chains run for 5,000,000 generations sampling every 50,000 generations after 1,000,000 generations of burnin. Bayesian runs were checked for proper mixing and convergence using standard diagnostics. Numbers above branches represent Bayesian posterior probabilities (PP > 0.7 are displayed). Cladograms show relationships among taxa while branch lengths are displayed in the inset phylograms. Branches are drawn proportional to the number of substitutions per site (see scale bar). Blue, green and red squares represent populations from the Balearic Islands, the southern (the Cadiz province) and southeast (the Almeria province) of the Iberian Peninsula, respectively. Species and population codes are shown in Table 1.

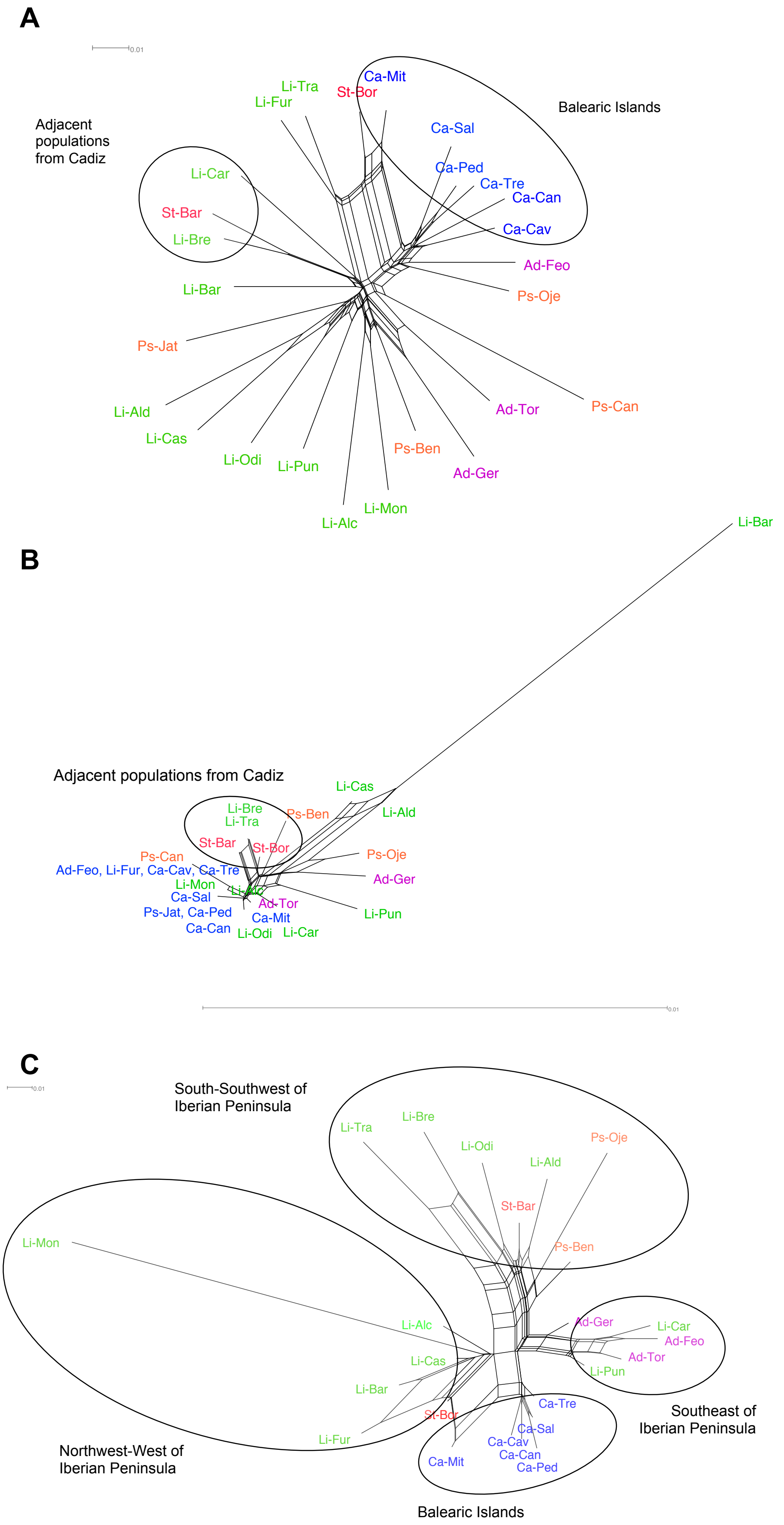

Figure S2: Phylogenetic relationships within the Section *Psammophilae* using phylogenetic networks. Neighbor-Net phylogenetic network for the cpDNA (A),

mtDNA (B) and nrDNA (C) genomes. Circles indicate the main groups either taxonomically or biogeographically linked discussed in the text. Each species is represented by different colors (*S. adscendens* = pink, *S. cambessedesii* = blue, *S. littorea* = green, *S. psammitis* = orange, *S. stockenii* = red). Species and population codes are shown in Table 1.

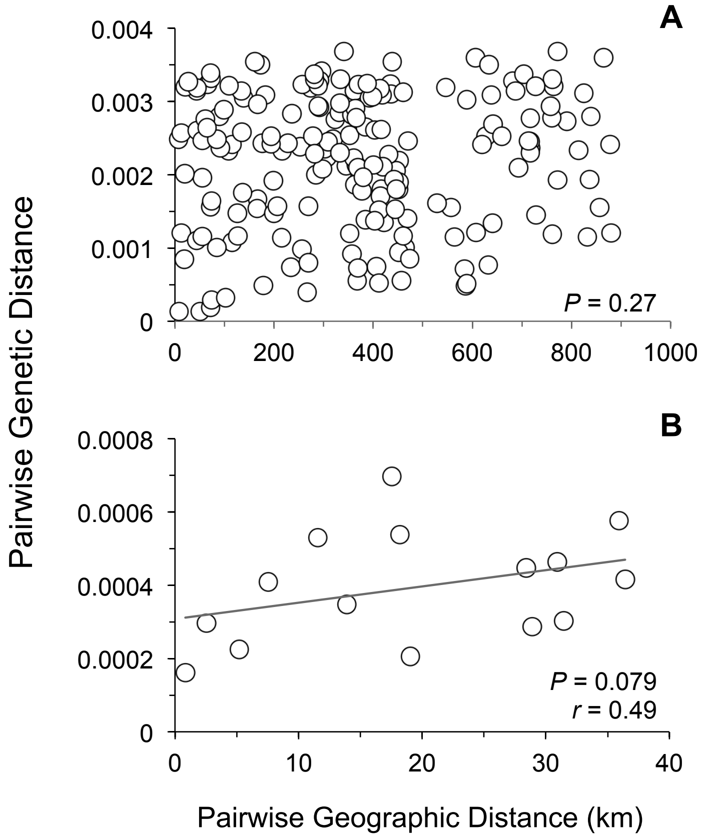

Figure S3: Partial Mantel test results. Isolation by distance detected by comparing pairwise genetic and geographic distance among mainland (A) and island populations (B).

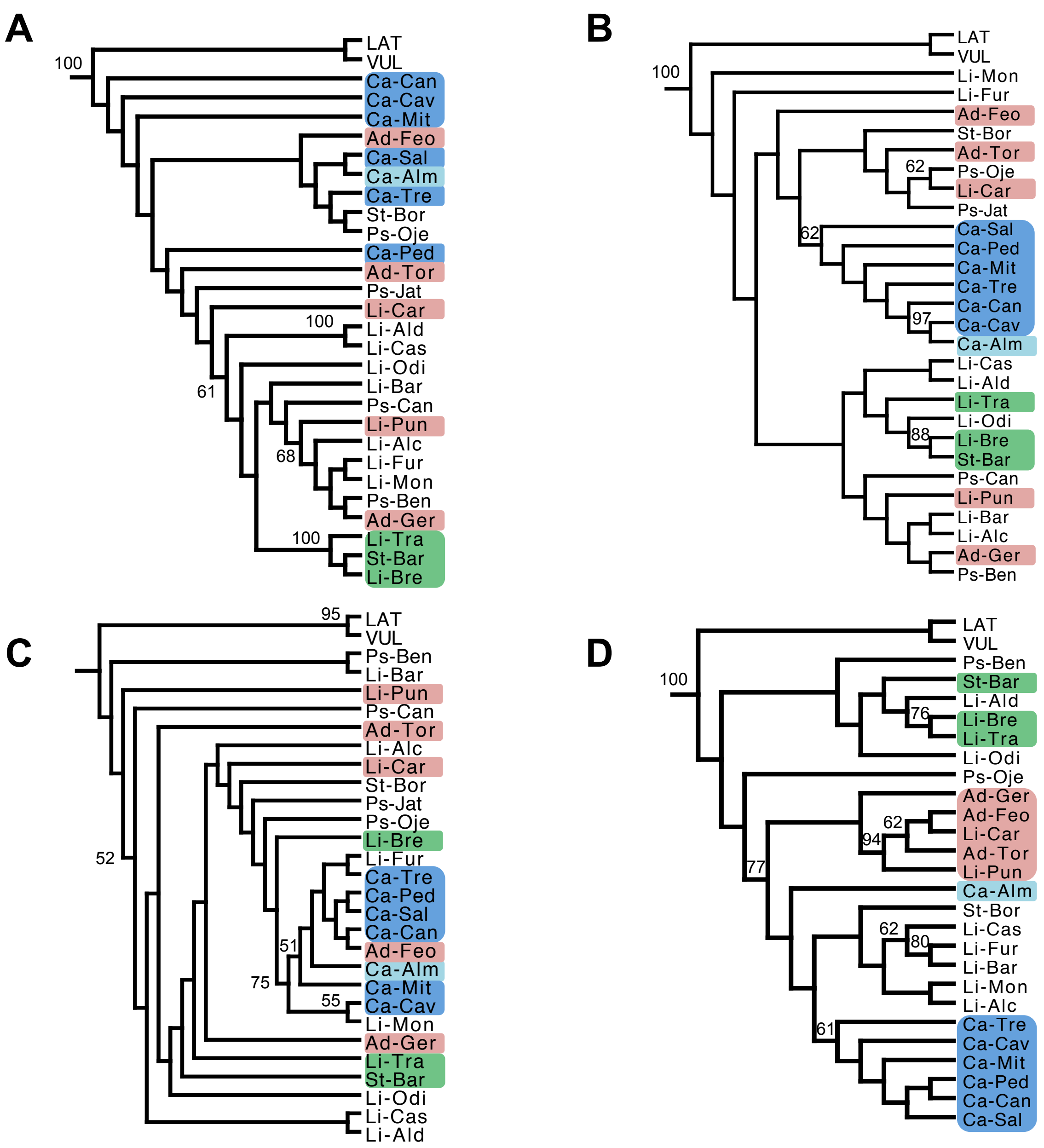

Figure S4: Phylogenetic relationships between mainland and island populations of *S. cambessedesii* using maximum likelihood estimation. Phylogenetic relationships were determined from the *trnK* (A) and *ycf1* chloroplast fragments (B), the *atp1* mitochondrial fragment (C) and the nuclear *ITS* region (D). Sequences were aligned, rooted with two outgroups and analyze under ML phylogenetic methods (RAxML). Numbers above branches represent bootstrap support (BS > 50 are displayed). Given that we want to show relationships among taxa, only cladograms are displayed. Blue, light-blue, green and red squares represent populations of *S. cambessedesii* from the Balearic Islands, the mainland *S. cambessedesii* population (Almenara), the southern (Cadiz province) and southeast (Almeria province) of the Iberian Peninsula, respectively. Species and population codes are shown in Table 1.
