# Supplementary material for: Whole Plastome Sequencing Within Silene Section Psammophilae Reveals Mainland Hybridization and Divergence With the Balearic Island Populations
Source: Front Plant Sci. 2019 Nov 15;10:1466. doi: 10.3389/fpls.2019.01466 (PMC6872646; doi:10.3389/fpls.2019.01466)
Supplement: Supplementary file 2 [file Table_1.docx]

| **Supporting Information**  Additional Supporting Information may be found in the online version of this article:  **Table S1** Sequencing results and cpDNA, mtDNA and nrDNA assemblies’ information. | | | | | | | | | | | | |
| --- | --- | --- | --- | --- | --- | --- | --- | --- | --- | --- | --- | --- |
|  |  | **cpDNA** | | |  | **mtDNA** | | |  | **nrDNA** | | |
| **Pop.** | **Total reads** | **Reads mapped** | **Average depth (± SD)** | **Assembly length (bp)** |  | **Reads mapped** | **Average depth (± SD)** | **Assembly length (bp)** |  | **Reads mapped** | **Average depth (± SD)** | **Assembly length (bp)** |
| Ad-Feo | 912514 | 125766 | 152  (± 57.5) | 153088 |  | 16680 | 12.4  (± 38.2) | 254399 |  | 25850 | 1067.5  (± 1328.4) | 12648 |
| Ad-Ger | 1443492 | 127520 | 147.8  (± 53.9) | 153180 |  | 27427 | 19.9  (± 34.7) | 254517 |  | 43490 | 1709.1  (± 2010.6) | 11916 |
| Ad-Tor | 1901158 | 169088 | 192  (± 67.4) | 153670 |  | 23149 | 16.5  (± 42.2) | 254545 |  | 63728 | 2720.5  (± 3141.2) | 13821 |
| Ca-Can | 1957440 | 99741 | 115.5  (± 54.5) | 153116 |  | 11052 | 8.0  (± 27.7) | 254314 |  | 67333 | 3409.2  (± 5734.3) | 11111 |
| Ca-Mit* | 28044404 | 3160806 | 1071.5  (± 758.7) | 158007 |  | 387679 | 131.8  (± 674.4) | 261291 |  | 716235 | 5524.1  (± 794.4) | 6769 |
| Ca-Sal | 2192580 | 74050 | 84.7  (± 38.1) | 153151 |  | 10728 | 7.8  (± 31.8) | 254408 |  | 108861 | 4328.8  (± 5425.2) | 14874 |
| Ca-Cav | 1693310 | 103010 | 119.3  (± 48.0) | 153183 |  | 14412 | 10.3  (± 32.8) | 254364 |  | 11437 | 2093.9  (± 2595.8) | 11437 |
| Ca-Ped | 2116526 | 140804 | 165.6  (± 64.5) | 153332 |  | 20622 | 15.0  (± 46.0) | 254476 |  | 88583 | 3456.0  (± 5116.7) | 14367 |
| Ca-Tre | 2226176 | 161616 | 188.7  (± 76.8) | 153274 |  | 21219 | 15.4  (± 50.0) | 254424 |  | 81022 | 3205.2  (± 5020.4) | 14010 |
| Li-Mon | 1033704 | 100284 | 119.8  (± 41.1) | 153957 |  | 9040 | 6.6  (± 29.2) | 254301 |  | 38639 | 1486.1  (± 2258.8) | 13208 |
| Li-Cas | 1010616 | 100058 | 123.7  (± 37.0) | 153145 |  | 9477 | 7.1  (± 25.7) | 254199 |  | 36531 | 1647.4  (± 2461.4) | 13036 |
| Li-Alc | 818956 | 67247 | 77.2  (± 28.4) | 153084 |  | 6444 | 4.6  (± 17.0) | 254141 |  | 26562 | 885.8  (± 1235.5) | 10925 |
| Li-Fur* | 26939945 | 5582746 | 1863.1  (± 774.4) | 157746 |  | 658036 | 197.2  (± 952.7) | 261066 |  | 984642 | 7584.7  (± 930.8) | 6878 |
| Li-Car | 1119274 | 85877 | 104.5  (± 46.5) | 153254 |  | 10410 | 7.9  (± 27.4) | 254416 |  | 51646 | 1999.5  (± 2094.2) | 13870 |
| Li-Pun | 1034724 | 182238 | 217.3  (± 80.2) | 153463 |  | 19177 | 14.3  (± 52.4) | 254465 |  | 33416 | 1220.9  (± 1451.9) | 13014 |
| Li-Bre | 1143368 | 156483 | 177.6  (± 63.7) | 153209 |  | 12931 | 9.1  (± 40.4) | 254159 |  | 22656 | 906.0  (± 1559.2) | 10502 |
| Li-Tra* | 24268786 | 6255056 | 2064.8  (± 564.2) | 157288 |  | 484344 | 145.9  (± 861.4) | 259665 |  | 504931 | 3896.4  (± 486.4) | 6736 |
| Li-Odi | 1894530 | 226646 | 257.3  (± 128.1) | 153287 |  | 19142 | 13.7  (± 48.7) | 254393 |  | 45254 | 2542.4  (± 4444.6) | 11936 |
| Li-Ald | 983688 | 101633 | 123.8  (± 46.0) | 153179 |  | 8356 | 6.1  (± 27.3) | 254141 |  | 13484 | 1494.4  (± 1780.3) | 13484 |
| Li-Bar | 1691660 | 118025 | 138.4  (± 61.5) | 153135 |  | 13198 | 9.8  (± 40.1) | 254305 |  | 90189 | 3485.3  (± 5129.7) | 14668 |
| Ps-Can | 791090 | 42858 | 49.9  (± 24.1) | 153124 |  | 5113 | 3.8  (± 10.1) | 254097 |  | 32139 | 1477.4  (± 1878.8) | 13264 |
| Ps-Jat | 1804136 | 154448 | 186.3  (± 69.3) | 153790 |  | 14265 | 10.6  (± 38.5) | 254594 |  | 49370 | 1566.7  (± 2167.4) | 12607 |
| Ps-Ben | 1991914 | 173362 | 195.7  (± 83.8) | 153224 |  | 16717 | 11.9  (± 46.2) | 254290 |  | 52904 | 1756.8  (± 1773.7) | 14150 |
| Ps-Oje | 1410414 | 107673 | 128.6  (± 56.3) | 153310 |  | 12273 | 9.2  (± 28.2) | 254436 |  | 41868 | 1828.0  (± 2241.9) | 13939 |
| St-Bar | 1259426 | 135176 | 155.9  (± 61.5) | 153278 |  | 14016 | 10.2  (± 34.1) | 254335 |  | 59767 | 4130.0  (± 6968.1) | 12501 |
| St-Bor* | 24389137 | 3379567 | 1136.7  (± 612.5) | 157927 |  | 435850 | 162.1  (± 1171.9) | 260777 |  | 839968 | 6472.8  (± 867.5) | 6831 |
| Population codes are shown in Table 1; * indicates samples from SE 50 bp sequencing. | | | | | | | | | | | | |

| **Table S2** List of GenBank accessions numbers for cpDNA, mtDNA and nrDNA sequences. | | |
| --- | --- | --- |
|  |  | GenBank accession numbers |
| **cpDNA** |  | MN365968 - MN365993 |
| **mtDNA** | *atp1* | MN334914 - MN334939 |
|  | *atp4* | MN334784 - MN334809 |
|  | *atp6* | MN334810 - MN334835 |
|  | *cob* | MN334836 - MN334861 |
|  | *cox3* | MN334862 - MN334887 |
|  | *nad9* | MN334888 - MN334913 |
| **nrDNA** |  | MN325944 - MN325960 |
